# Insights into the Effect of Magnetic Confinement on the Performance of Magnetic Nanocomposites in Magnetic Hyperthermia and Magnetic Resonance Imaging

**DOI:** 10.1021/acsanm.2c03537

**Published:** 2022-11-07

**Authors:** Stefania Scialla, Nuria Genicio, Beatriz Brito, Malgorzata Florek-Wojciechowska, Graeme J. Stasiuk, Danuta Kruk, Manuel Bañobre-López, Juan Gallo

**Affiliations:** † Advanced (Magnetic) Theranostic Nanostructures Lab, International Iberian Nanotechnology Laboratory, Av. Mestre José Veiga s/n, 4715-330Braga, Portugal; ‡ Department of Imaging Chemistry and Biology, School of Biomedical Engineering and Imaging Sciences, King’s College London, Strand, LondonSE1 7EH, U.K.; § School of Life Sciences, Faculty of Health Sciences, University of Hull, Cottingham Road, HullHU6 7RX, U.K.; ∥ Department of Physics and Biophysics, Faculty of Food Science, University of Warmia & Mazury in Olsztyn, Oczapowskiego 4, 10-719Olsztyn, Poland

**Keywords:** magnetic nanocomposites, magnetic particle interactions, magnetic resonance imaging, magnetic hyperthermia, theranostics

## Abstract

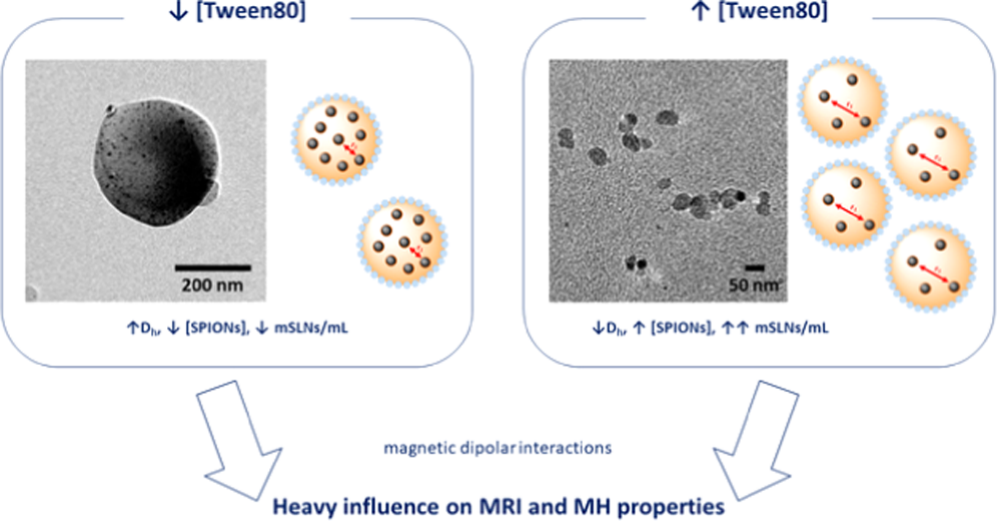

The combination of superparamagnetic iron oxide nanoparticles (SPIONs) and lipid matrices enables the integration of imaging, drug delivery, and therapy functionalities into smart theranostic nanocomposites. SPION confinement creates new interactions primarily among the embedded SPIONs and then between the nanocomposites and the surroundings. Understanding the parameters that rule these interactions in real interacting (nano)systems still represents a challenge, making it difficult to predict or even explain the final (magnetic) behavior of such systems. Herein, a systematic study focused on the performance of a magnetic nanocomposite as a magnetic resonance imaging (MRI) contrast agent and magnetic hyperthermia (MH) effector is presented. The effect of stabilizing agents and magnetic loading on the final physicochemical and, more importantly, functional properties (i.e., blocking temperature, specific absorption rate, relaxivity) was studied in detail.

## 1 Introduction

Hybrid nanocomposites are among the frontier multifunctional platforms. They combine structural components of two or more (organic and inorganic) materials, benefiting from the synergistic properties of the individual components. In this context, hybrid magnetic nanosystems have enticed researchers’ attention as smart remotely controlled platforms, able to pool together diagnostic, monitoring, and combinatorial therapy features. Over the last decades, advances in the field of theranostics (therapy plus diagnosis) have brought a variety of organic/inorganic hybrid nanocomposites with a high level of integration and complexity, featuring tunable functions strictly related to well-defined architectures.^1−5^ Nevertheless, the integration of diagnostic and therapeutic functionalities in a smart all-in-one platform in the form of hybrid nanocomposites remains a big challenge.

Solid lipid nanoparticles (SLNs) represent a valid alternative to liposomes, emulsions, and polymeric nanostructures because of their high physical stability and ease of preparation.^6,7^ SLNs consist of a biodegradable and biocompatible lipid nucleus surrounded by surfactants to enhance their colloidal stability.^8^ The choice of lipids and surfactants and their concentration impact the quality of the SLN dispersion in terms of particle size, long-term stability, drug loading efficacy, and drug release.^9,10^

Superparamagnetic iron oxide nanoparticles (SPIONs) have become benchmark inorganic materials because of their superparamagnetic properties (high saturation magnetization, null coercivity, and anisotropy)^11^ and clinic introduction. In fact, they can act as negative contrast-enhancing agents for magnetic resonance imaging (MRI)^12,13^ by shortening the transverse relaxation time (*T*_2_) of water protons in nearby tissues. This translates into a significant increase in the transverse relaxivity (*r*_2_) and a darker (hypointense) signal in the MRI images.^14^ Moreover, SPIONs can also act as heating effectors by locally increasing the temperature upon exposure to an alternating magnetic field (AMF). The heat released arises from the relaxation of the magnetic moment within the particle (Néel relaxation) or the rotation of the particle itself (Brownian relaxation).^15−17^ The contrast and heating behavior of SPIONs significantly depends on not only their inherent features, like size, crystal structure, and morphology, but also their spatial distribution, aggregation state, physical confinement, and surrounding environment.^17−21^

SPION confinement within a nonmagnetic counterpart can result in cluster formation, where the mean interparticle distance is reduced and becomes relevant in the magnetic collective behavior of the nanosystem. SPION confinement within organic matrixes may change the superparamagnetic regime, translating into positive or negative effects on MRI and magnetic hyperthermia (MH) performance. Several studies have shown that SPION confinement results in a superior *r*_2_ in the nearby water protons,^5,22−24^ facilitating their detection by MRI and decreasing image artifacts. For instance, the relaxivity of SPIONs confined in the inner core of lipid nanosystems was reported to be 2–5-fold higher than that of single noninteracting SPIONs (*r*_2_ ≈ 200–900 mM^–1^·s^–1^ at 1.41 T vs *r*_2_ = 49.5 mM^–1^·s^–1^ at 1.5 T).^1,5,25^ This variation might be presumably ascribed to differences in SPION clusters’ magnetic moment and concentration, and to the hydrophobic nature of the lipid core preventing the diffusion of water molecules toward SPIONs embedded in the lipid matrix.^26^ Hence, the interaction between the water protons and the magnetic field generated by the SPIONs extends over time, translating into enhanced relaxivity properties.^27^ In macroscopic systems such as hydrogels, there has been no agreement with the presented results. For example, a modest increase in *r*_2_ (up to 134 mM^–1^·s^–1^ at 3.0 T) was observed by incorporating SPION nanoparticles in a chitosan-based hydrogel. This enhancement was explained by the particle aggregation effect. As the viscosity increases, magnetic nanoparticles may display a more compact arrangement in the confined space. Hence, the particle magnetic moment get strengthened, directly improving the transverse relaxometric performance.^28^ On the contrary, SPIONs confined into self-assembled dehydrodipeptide-based hydrogels resulted in up to a fourfold reduction of *r*_2_ compared to free SPIONs (*r*_2_ ≈ 30–44 mM^–1^·s^–1^ vs *r*_2_ ≈ 120 mM^–1^·s^–1^ at 3.0 T). These observed low *r*_2_ relaxivities are probably ascribed to the restricted water diffusion throughout the hydrogel matrix.^29^

Very few works have explored the effect of magnetic dipole–dipole interactions, associated with particle surface morphologies, structures, and concentrations, on the magnetic hyperthermia behavior, measured through the specific absorption rate (SAR).^16,17,30−36^ It was reported that the heating capability of confined magnetic nanosystems is generally twofold higher (SAR > 200 W·g_Fe_^–1^ at 869 kHz, 25 mT, 1 mg_Fe_·mL^–1^) than that of single SPIONs with similar core size and magnetic composition (i.e., Ferumoxytol SAR ≈ 250 W·g^–1^, Resovist SAR ≈ 106 W·g^–1^, Feridex SAR ≈ 115 W·g^–1^).^1,5,37^ However, several works have highlighted that the SPION clusters may cause demagnetizing effects, resulting in a drastic reduction of the encapsulated SPIONs’ heating performance^21,38^ as a consequence of heterogeneous particle aggregation and annihilation of the Brownian contribution to the total heating performance.^39^ A significant decrease of SAR was, in fact, reported by Carvalho et al. by increasing SPIONs loading into self-assembled dehydropeptide-based hydrogels.^29^ Similarly, also Ribeiro et al. proved that the SAR of SPION-confined xanthan gum hydrogel was remarkably lower than that of free SPIONs (SAR ≈ 100 W·g^–1^ vs SAR ≈ 400 W·g^–1^, measured at 20 mT and 869 kHz).^40^ This reduction was caused by SPIONs being physically constrained within the polymeric network of the hydrogel, resulting in a drastic reduction of the Brownian relaxation contribution to the overall relaxation mechanism. Hence, the relationship between the SPIONs’ heating capacity and their dipole intereactions is not well established yet.

Herein, we present a systematic study on the functional performance of one such confined magnetic system. The incorporation of magnetic iron oxide nanoparticles into wax matrices enables the preparation of theranostic probes with great biomedical potential. The effect of surfactant concentration and SPION loading into magnetic solid lipid nanoparticles (mSLNs) was investigated, particularly in terms of their MRI and MH properties. Variations in these design parameters allow us to obtain a series of configurations where the embedded SPIONs showed differences in their spatial distribution within the lipid matrix, leading to a controlled aggregation phenomenon, which reflects on some relationship between the intra-/interparticle interactions and magnetic performance of the final magnetic nanocomposite systems.

## 2 Materials and Methods

### 2.1 Chemicals

Iron(II) chloride tetrahydrate (FeCl_2_·4H_2_O), iron(III) chloride hexahydrate (FeCl_3_·6H_2_O), oleic acid (OA, ≥ 99%), ammonium hydroxide (NH_4_OH), hexane (analytical grade), chloroform (CHCl_3_, analytical grade), and polyoxyethylene sorbitan monooleate (Tween80) were purchased from Sigma-Aldrich. Carnauba wax was a kind gift from KosterKeunen Holland BV. Hydrochloric acid (HCl, 37% v/v) was purchased from Fisher Chemicals. Milli-Q water (ultrapure) was used throughout all tests.

### 2.2 Synthesis of Superparamagnetic Iron Oxide Nanoparticles

**Figure 1:**
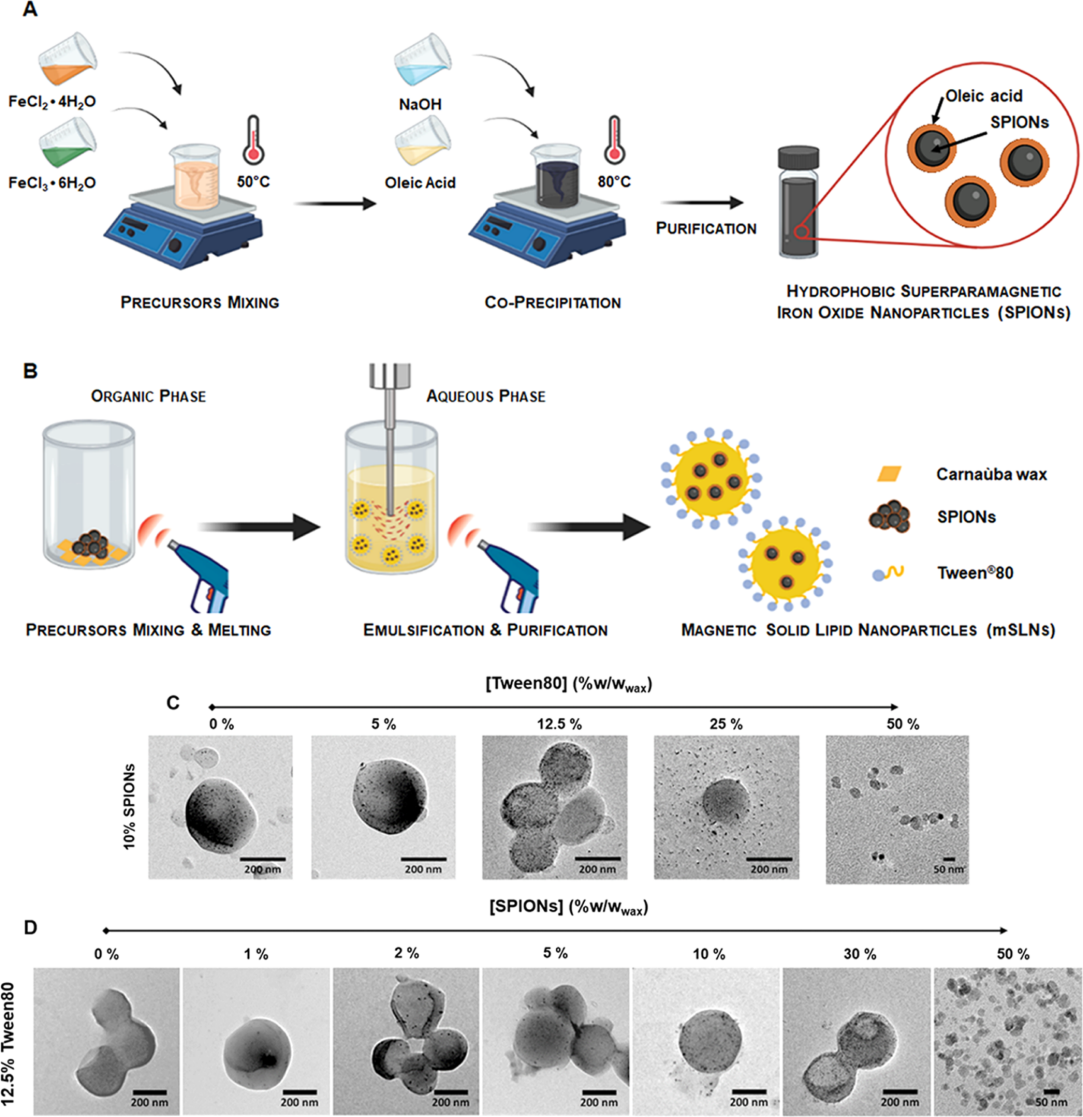
(A) Schematic representation of the hydrophobic SPION synthesis by the coprecipitation method. (B) Representative scheme of the melt-emulsification solvent evaporation method used for mSLN preparation. Representative transmission electron microscopy (TEM) micrographs of the different mSLN formulations investigated in this study showing the effect of (C) Tween80 concentration (increasing from left to right) [the scale bar represents 200 nm except for 50% Tween80, in which the scale bar represents 100 nm] and (D) SPION loading (increasing from left to right) [the scale bar represents 200 nm except for 50% SPIONs, in which the scale bar represents 50 nm].

Hydrophobic magnetite nanoparticles coated with oleic acid (SPIONs) were prepared by a high-yield coprecipitation adapted method previously described.^41^ Briefly, 9.2 g of FeCl_2_·4H_2_O and 15 g of FeCl_3_·6H_2_O were dissolved in 250 mL of Milli-Q water and stirred for 10 min at 50 °C. After 10 min, 12 M NH_4_OH (30 mL) was added to the solution, resulting in a dark iron oxide precipitate. OA (2.5 mL) was then added, and the mixture was heated at 80 °C for 1 h. Afterward, the mixture was cooled down to room temperature. Excess NH_4_OH and OA were removed by magnetic separation of the precipitate OA-coated iron oxide by an external magnet, followed by redispersion in fresh solvent. The washing procedure was repeated two times with Milli-Q water and one time with hexane. Finally, SPIONs were dispersed in CHCl_3_ and stored at 4 °C until further use. A scheme of the synthesis is reported in Figure 1A.

### 2.3 Synthesis of Magnetic Solid Lipid Nanoparticles

A modified emulsification solvent evaporation method was used to synthesize magnetic solid lipid nanoparticles (mSLNs).^5^ In the preparation of the organic phase (OP), different amounts of SPIONs (0, 0.5, 1, 2, 5, 10, 30, 50% w/w_wax_) were added to 100 mg of wax. This pre-OP was melted by heating, and the aqueous phase (AP), consisting of Tween80 at different concentrations (0, 5, 12.5, 25, 50% w/w_wax_) in Milli-Q water (*V*_tot_ = 2.25 mL), was added to the OP and sonicated under high-power ultrasounds (Branson W-450D Sonifier ) for 2 min at a 25% power and 20 s working intervals (20 s on, 20 s off), keeping the system warm. After sonication, the formulation was rapidly cooled down using an ice bath and then centrifuged (3000 rpm, 10 min) to eliminate unreacted components and large wax aggregates, the pellets were discarded, and the supernatants were stored. Nonmagnetic solid lipid nanoparticles were also synthesized and used as control. A scheme of the process here-detailed is reported in Figure 1B.

### 2.4 Physicochemical Characterization of the mSLNs

SPION and mSLN morphology was explored by transmission electron microscopy (TEM, JEM 2100 HT). Bright-field TEM micrographs, acquired at 200 keV, were collected by a “OneView” 4k × 4k charge-coupled device (CCD) camera. The samples were prepared by dropping 7 μL of SPIONs diluted in CHCl_3_ and mSLNs diluted in Milli-Q water onto a carbon-coated Cu grid followed by evaporation of the solvent under vacuum at room temperature. The micrographs were acquired at different magnifications (10–500k×) in at least five regions of interest for each sample; *n* > 300 nanoparticles were analyzed for SPION size distribution. TEM micrographs were elaborated using ImageJ 1.50c (Fiji) software.

The iron oxide phase formation and crystallographic state of the SPIONs were investigated using an X’Pert PRO diffractometer (PANalytical) set at 45 kV and 40 mA and equipped with Cu Kα radiation (λ = 1.541874 Å) using the Bragg–Brentano geometry of 2θ scanning range of 25–65° and a scanning speed of 0.006°·s^–1^. The X-ray diffraction (XRD) patterns were matched to the Crystallography Open Database (COD using High Score software package, PANalytical). The inorganic and organic contents of SPIONs were determined by thermogravimetric analysis (TGA, SDTA 851e balance, Mettler Toledo). Dynamic light scattering (DLS, Horiba nanoPartica SZ-100) was employed to measure the hydrodynamic diameter (*D*_h_), polydispersion index (PDI), and surface charge (ξ-potential) of the mSLNs. mSLNs were diluted in Milli-Q water (1:50, v/v) to obtain a signal of 100–500 kcps measured using a carbon electrode cell. The system operates in backscattering mode with a He–Ne laser beam (λ = 532 nm) at 25 °C; the scattering angle was set at 173° with a stabilization time of 180 s and considering the refractive index of the magnetite of 2.420. The results are shown as the average of all independent measurements (3–5 times) ± the standard error mean (s.e.m) by SZ-100 software. Nanoparticle tracking analysis (NTA) was performed with a Malvern Panalytical NanoSight LM10 (Spectris Inc.) equipped with a scientific complementary metal oxide semiconductor (CMOS) camera, a blue laser module (405 nm), and NanoSight software (version Build 3.4.003). A 1 mL disposable syringe was used to inject the samples into the instrument chamber. mSLNs were diluted in Milli-Q water until a concentration between 1 × 10^4^ and 1 × 10^6^ particles·mL^–1^, and the video data for NTA measurements were collected for 60 s at room temperature, repeated three times for each sample, with manual shutter and gain adjustments. Detected tracks were then translated into the concentration of particles (number of particles·mL^–1^). The iron (Fe) concentration in the SPION and mSLN samples was determined by inductively coupled plasma emission spectroscopy (ICP-OES, ICPE-9000 Multitype ICP Emission Spectrometer, Shimadzu). Samples were prepared by digesting a small volume (30–100 μL) of SPIONs and mSLNs in 1 mL of HCl (37% v/v) overnight and diluting them with Milli-Q water (up to 10 mL total volume). Samples were filtered by 0.45 μm cutoff nylon hydrophilic-based syringe filters (Millipore) before the analysis. Measurements were repeated three times, and the results were expressed as mean ± s.e.m. The Fe concentration was converted in Fe_3_O_4_, assuming that the theoretical amount of Fe in Fe_3_O_4_ is 72.3%. The encapsulation efficiency of SPIONs was calculated according to eq 1
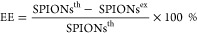
1where SPIONs^th^ is the theorical amount of magnetite (expressed as % w/w_wax_) used to prepare the different mSLN formulations and SPIONs^ex^ is the experimental amount of magnetite (expressed as % w/w_wax_) calculated from the corresponding Fe content quantified by ICP-OES.

Field-dependent magnetization curves of SPIONs were recorded using a superconducting quantum interference device magnetometer (SQUID-VSM, Quantum Design) in a magnetic field ranging from −20 to +20 kOe at 5 and 300 K (room temperature). Zero-field-cooled and field-cooled (ZFC–FC) magnetization curves of mSLNs were recorded in a SQUID-VSM magnetometer over the temperature range 2–300 K and under an applied magnetic field of 100 Oe. For sample preparation, a small volume of SPION and mSLN dispersions (50 μL) was placed in a polypropylene (PP) mold, dried in a vacuum chamber, introduced in a standard brass sample holder, and attached to a measuring rod. Both PP molds and brass sample holders were provided by Quantum Design. The magnetization units were expressed as emu per gram of magnetic sample.

### 2.5 Hyperthermia Measurements

Magnetic heating curves were acquired with a DM 100 System (nB nanoScale Biomagnetics). Measurements were performed by introducing 500 μL of each mSLN (Fe = 100 μg·mL^–1^) formulation into a 1.5 mL glass vial, which was placed at the midpoint of a water-cooled copper coil. Then, a three-step protocol was applied: (i) *f* = 0 kHz, *t* = 15 min, *H* = 0 mT; (ii) *f* = 869 kHz, *t* = 15 min, *H* = 20 mT; and (iii) *f* = 0 kHz, *t* = 15 min, *H* = 0 mT. When applying the oscillating magnetic field, the temperature was monitored as a function of time by an integrated optical fiber-based temperature measurement system. Specific absorption rates (SAR, W·g_Fe_^–1^) were calculated according to eq 2
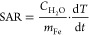
2where *C* is the specific heat capacity of the medium (assumed equal to that of water, *C*_H_2_O_ = 4185 J·L^–1^·K^–1^), *m* is the Fe concentration (g_Fe_·L^–1^) of the magnetic material in solution, and d*T*/d*t* is the slope of the initial linear section of the temperature versus time curve. The SAR values were determined by the initial slope method. For the calculations, the initial 50 s after the application of the oscillating magnetic field were considered in the linear fitting.

### 2.6 Relaxivity Measurements

Relaxation times were measured using a Minispec benchtop relaxometer (mq 60, Bruker, *B*_0_ = 1.41 T) operating at 60 MHz. Samples with Fe concentrations between 0 and 0.15 mM were preheated at 37 °C and kept at this temperature during the experiments. *T*_1_ (s) and *T*_2_ (s) relaxation times were measured using standard saturation recovery (SR) and Carr–Purcell–Meiboom–Gill (CPMG) sequences, respectively. The longitudinal (*r*_1_, mM^–1^·s^–1^) and transverse (*r*_2_, mM^–1^·s^–1^) relaxivities of the mSLNs samples were calculated by fitting the curves of longitudinal *T*_1_^–1^ and transverse *T*_2_^–1^ (s^–1^) relaxation rates plotted as a function of Fe concentration (mM). ^1^H spin–lattice relaxation rates were also measured in the frequency range from about 5 kHz to 10 MHz using a SpinMaster2000 relaxometer (Stelar srl).

### 2.7 Magnetic Resonance Imaging Studies

MR imaging was performed using a 3 T horizontal bore MR Solutions Benchtop scanner equipped with 48 G·cm^–1^ actively shielded gradients. To image the samples, a 56 mm diameter quadrature bird-cage coil was used in transmit/receive mode. For the MRI phantom measurements, mSLN samples were prepared by fixing first the Fe concentration (100 μM) and by fixing in the second measurement the number of nanoparticles (10^12^ particles·mL^–1^). About 300 μL of each sample was placed on a custom-printed PLA well plate, which was then placed at the center of the coil. *T*_2_-weighted images were acquired using the fast spin echo (FSE) sequence with the following parameters: TE = 11 ms, TR = 12,000 ms, NA = 32. MRI images of phantoms were acquired with an image matrix of 256 × 252, FOV of 60 × 60 mm, six slices with a slice thickness of 0.5 mm, and a slice gap of 0 mm. Image analysis was performed using ImageJ software (http://imagej.nih.gov/ij).

## 3 Results and Discussion

### 3.1 Superparamagnetic OA-Coated Iron Oxide Nanoparticles

Monocrystalline iron oxide nanoparticles coated with oleic acid (SPIONs) were synthesized by a high-yield, easily scalable, two-step coprecipitation method,^41^ as illustrated in Figure 1A. This method provided hydrophobic SPIONs with high colloidal stability in nonpolar solvents, thanks to the chemical interactions between the carboxylic groups of OA and iron atoms on the surface of the inorganic core, orienting the hydrophobic tails of OA outward. OA is widely used as coating ligand for iron oxide nanoparticles;^43^ its use minimizes oxidation and aggregation effects and, in this case, ensures chemical compatibility of the SPIONs with the organic lipid matrix used for the production of final magnetic nanocomposites. SPIONs were morphologically characterized using TEM. Micrographs (Figure SI 1A, inset) revealed a pseudospherical shape with a monodispersed distribution centered at 9.7 ± 0.1 nm. The size distribution presents a bell shape that can be fitted to a Gaussian equation, characteristic of this kind of magnetic particles (Figure SI 1A).^44^ The crystal structure of the SPIONs was determined by XRD (Figure SI 1B), displaying a single phase with inverse spinel crystallographic structure in accordance with Bragg’s reflections of pure magnetite (Fe_3_O_4_) reported in COD 96-900-5838. The field-dependent magnetization curves (Figure SI 1C), normalized to the mass of vacuum-dried SPIONs, showed that they behave as superparamagnetic materials, in which both remnant magnetization and coercivity are quasi-zero (*M*_r_ = 3.3 emu·g^–1^; *H*_c_ = 0.04 kOe, respectively) despite an extrinsic contribution to the coercivity from the magnetic flux trapped in the superconducting coil. The SPIONs present high saturation magnetization (*M*_s_ = 67 emu·g^–1^), although, as expected, significantly lower than the value of bulk magnetite (*M*_s_ = 92 emu·g^–1^) due to a nonuniform distribution of spins on the nanoparticle surface (spin canting), as previously reported.^45^ It is important to highlight that the *M*_s_ value was higher than that of Feridex (*M*_s_ = 45 emu·g^–1^), a SPION-based formulation, approved by the Food and Drug Administration (FDA) as an MRI contrast agent.^46^ ZFC–FC magnetization curves revealed a *T*_B_ of 170 K (Figure SI 1D), which represents the magnetic transition from a magnetically blocked state (below *T*_B_) to a superparamagnetic regime (above *T*_B_). All parameters related to SPIONs are summarized in Table SI 1.

### 3.2 Magnetic Solid Lipid Nanoparticles

A simple and scalable modified melt-emulsification method^5^ was followed to synthesize the series of hybrid nanocomposites (Figure 1B). This protocol is based on the homogenization of a lipid matrix within an aqueous phase. Specifically, the hydrophobic matrix consists of Carnauba wax, a natural wax approved by the FDA for end use in food, cosmetics, and pharmaceutical applications [21CFR184.1978 and 21CFR175.320], characterized by a high melting point of 82–86 °C. SPIONs were incorporated into the lipid nucleus, and a nonionic alkyl-phenolic surfactant, Tween80 (also approved by FDA for human use 21CFR172.840), was used as a stabilizer at the lipid/water interface. The use of surfactants plays a pivotal role in colloidal stability of the SLNs,^47,48^ influencing their surface properties.^49^ A series of mSLNs incorporating increasing amounts of SPIONs from 0 to 50% w/w_wax_ and using different concentrations of Tween80 from 0 to 50% w/w_wax_ were prepared.

### 3.3 Morphological and Physicochemical Characterizations

To get an insight into the mSLN structure, their morphology and SPION incorporation and distribution were evaluated by TEM (Figure 1C,D). The micrographs confirmed the successful incorporation of the SPIONs (clearly visible as smaller hypointense spots) within the larger spherical wax cores. In particular, at low SPION loadings (up to 2–5% w/w_wax_), these spots were randomly localized in the wax core, appearing as small nonaggregated magnetic nanoparticles. As the SPION loading increases (10–30% w/w_wax_), distribution of the spots was still observed within all of the spherical wax matrix, although with a slight preferential accumulation in the mSLN periphery as a consequence of the clustering effect of the magnetic cores (Figure 1D). At 50% Tween80, TEM micrographs show that the final nanostructures are not real nanocomposites but rather core–shell structures in which individual SPION cores are surrounded by a wax shell (Figure 1C).

**Figure 2:**
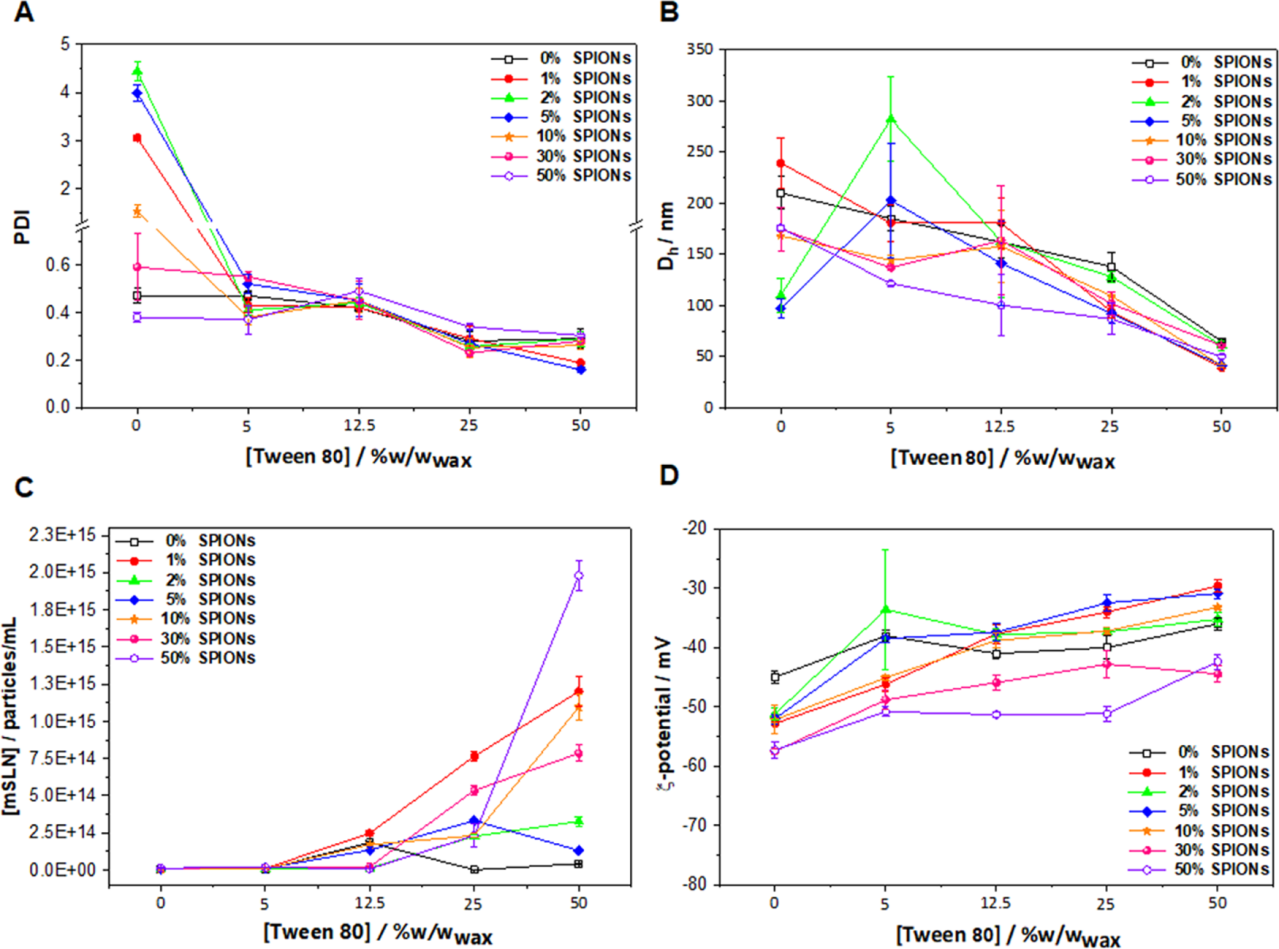
(A) Polydispersion index (PDI), (B) hydrodynamic diameter (*D*_h_) measured in Milli-Q water at 25 °C by DLS, (C) concentration of mSLNs as a function of Tween80 concentration measured in Milli-Q water at 25 °C by NTA, and (D) ξ-potential as a function of Tween80 concentration measured in Milli-Q water at 25 °C by DLS.

As already introduced, the nature and concentration of the surfactant play a key role in the physicochemical properties of lipid nanoparticles. High surfactant concentrations reduce the interfacial lipid/water tension, facilitating particle partition during emulsification.^50,51^ Tween80 (Polysorbate 80) is an amphipathic, nonionic synthetic surfactant, available as a chemical mixture of different fatty acid esters of polyoxyethylene sorbitan. The hydrocarbon chains of fatty acids provide the hydrophobic nature of the polysorbates, while the hydrophilic nature is provided by the ethylene oxide subunits.^52^ Surfactants particularly influence the particle size and size distribution, described by the polydispersion index (PDI). In drug delivery system applications using lipid carriers, a PDI lower than 0.4 is accepted as an indication of a moderately homogeneous population.^53−55^ In our case, the effect of surfactant concentration on the mSLN size and stability was studied by DLS analysis (Figure 2). It is worth noticing that even in the total absence of the surfactant (0% w/w_wax_), the formulations were produced, most likely due to the amphiphilic nature of some of the components of the wax. However, these formulations recorded the highest values of PDI, indicative of unstable colloidal stability, thus confirming the importance of the surfactant in the emulsification process (Figure 2A). A significant reduction in the PDI to values around 0.3 with increasing surfactant concentration from 0 to 25 (% w/w_wax_) was observed (Figure 2A), probably coming from a reduction of coalescence. At low surfactant concentrations, a complete mSLN surface coverage is most likely not achieved, resulting in larger PDI values and decreased nanocomposite stabilization and dispersion. At higher surfactant concentrations, the hydrocarbon chains fully cover the surface of the lipid cores, reducing surface tension and generating repulsive forces among the mSLNs.^50,56−58^ In our case, intermediate Tween80 concentrations (5–25% w/w_wax_) are able to provide efficient steric stabilization of the particles as described by the relatively low PDI values obtained. The highest Tween80 concentration tested (50% w/w_wax_) also provided good PDI values (Figure 2A), although the samples from this series belong to a family with a different structure, as shown below. Furthermore, as expected, a significant decrease in the hydrodynamic diameter of the mSLNs from 400 to 100 nm was observed by increasing the surfactant concentration (Figure 2B), as a result of a larger oil–water interface.^59−61^ Several studies have reported that there is a point where increasing the surfactant concentration further no longer decreases the particle size and may lead to an increase in size.^59,62,63^ This effect was not observed in this system, even at surfactant concentrations as high as 50% (Figure 2B). As expected, the decrease in size observed in this system is accompanied by the consequent increase in the number of particles (Figure 2C), as measured by nanoparticle tracking analysis.

The ξ_pot_ of the mSLNs showed highly negative values in the range between −60 and −20 mV (Figure 2D) due to the chemical composition of the wax and particularly Tween80. These values indicate a strong electrostatic repulsion between the mSLNs and high colloidal stability. Despite the increase of surfactant concentration resulting in a progressive decrease of the negative surface charge, this reduction was not significant enough to influence the colloidal stability.

**Figure 3:**
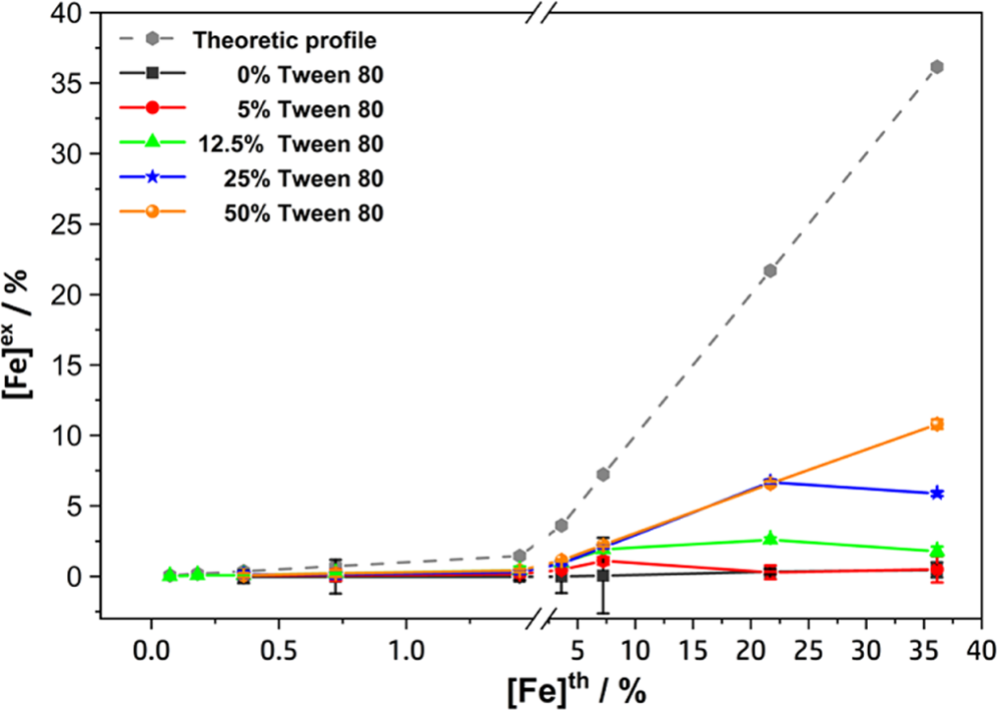
Experimental Fe content ([Fe]^ex^, %), quantified by ICP-OES after purification, as a function of the theoretical Fe content ([Fe]^th^, %) in the mSLN formulations prepared at different Tween80 concentrations.

The final experimental Fe concentration ([Fe]^ex^) in the different mSLN formulations was quantified by ICP-OES. The Fe concentration was converted to Fe_3_O_4_, assuming that the theoretical amount of Fe in Fe_3_O_4_ is 72.3%, and the SPION encapsulation efficiency within the mSLNs was then calculated according to eq 1. Even though the encapsulation efficiency of the SPIONs within the mSLNs was in the range of 69–100% (see Table SI 2), the final real SPION content in the different formulations was lower than the theoretical values due to the implementation of the purification (centrifugation) step after the synthesis to remove large wax aggregates. In Figure 3, the experimental amount of Fe ([Fe]^ex^, % w/w_wax_) is reported as a function of the theoretical one ([Fe]^th^, % w/w_wax_). Data analysis shows that at low initial SPION loadings (0.5–2%), no significant differences were observed in the final experimental amounts of Fe by increasing the surfactant concentration. However, at higher theoretical SPION loadings (5–25 and 50%), there was a significant increase in the experimental amount of Fe_3_O_4_ encapsulated with an increase in surfactant concentration.

### 3.4 Magnetic Characterizations

**Figure 4:**
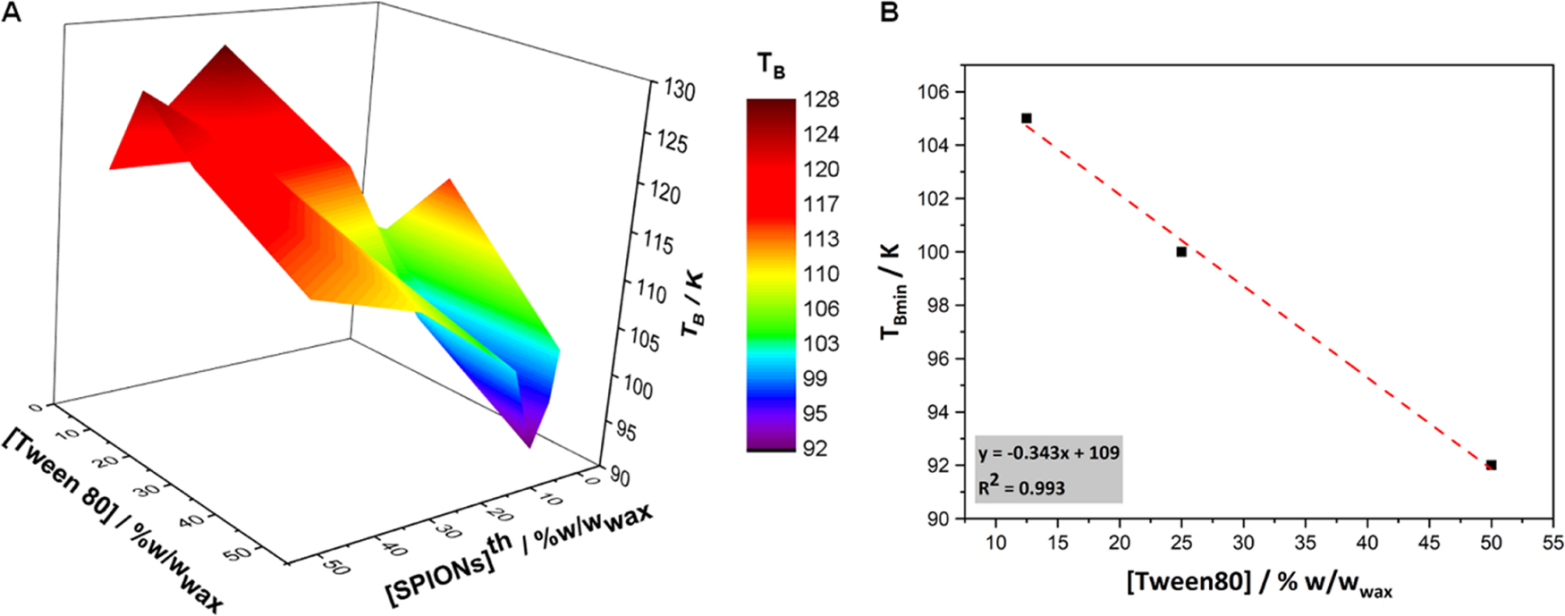
(A) Blocking temperature (*T*_B_) as a function of the theoretical SPION loadings and Tween80 concentrations used for the mSLN formulation preparation, extracted from the ZFC curves in the range of 2–300 K under an applied magnetic field of 100 Oe. (B) Dependence of *T*_Bmin_ on surfactant concentration, showing a linear relationship.

The magnetic response of mSLNs was studied by the blocking temperature (*T*_B_). Phenomenologically, *T*_B_ is defined as the maximum of the ZFC curve. As mentioned already in the Section 1, differences in *T*_B_ are closely related to the magnetic anisotropy, aggregation state, and volume distribution of the SPIONs. In a simple model based on two SPIONs with large anisotropies, if the NPs are far from each other, they act as two independent magnetic systems and the *T*_B_ decreases. When the distance between the two SPIONs decreases, the magnetic interactions increase and the SPIONs finally act as a single cooperating magnetic system, shifting the *T*_B_ toward higher values because of the increase in the total magnetic volume in the aggregated state.^24,64^ In our system, we observe two different magnetic regimes (Figures 4A and SI 2): (i) at low SPION loadings, *T*_B_ decreases with increasing SPION concentration, reaching a minimum that varies as a function of the concentration of Tween80; the higher the surfactant concentration, the higher the magnetic loading needed to minimize *T*_B_; (ii) at high SPION loadings (above *T*_Bmin_), a quasi-linear increase of *T*_B_ with magnetic loading up to 50% is observed. For the lowest percentage of Tween80 used (5%), a *T*_B_ maximum is reached at 30% SPION loading, above which a decrease in *T*_B_ is observed. This *T*_B_ maximum is not reached for the rest of the formulations. These results evidence that not only the Fe content of the mSLNs but also the Tween80 concentration rule the magnetic interaction of the mSLNs with the external magnetic field, eventually determining the value of *T*_B_. Interestingly, Figure 4B shows a linear dependence of *T*_Bmin_ on Tween80 concentration (the 5% Tween80 curve does not lead to a *T*_Bmin_ and as such it was not considered in the plot), so that the higher the Tween80 concentration, the lower the *T*_Bmin_. This decrease of *T*_Bmin_ with Tween80 concentration is in strong agreement with the observed reduction of *D*_h_, as well as with the increase of mSLN concentration, as the surfactant concentration increases in the series. As deduced from Figure 2, an increase of Tween80 concentration leads to a higher number of smaller mSLNs in the dispersion, meaning that SPIONs are more spatially distributed, avoiding particle aggregation and clustering effects and inducing a shift of *T*_B_ to lower temperatures. Overall, these different regimes could then be ascribed to the different packing fractions (ratio between the volume of SPIONs and the volume of the wax matrix in which they are encapsulated) among formulations. Above a SPION loading threshold, the magnetic packing density increase (confirmed by TEM) makes SPIONs magnetically couple among themselves, resulting in stronger dipolar interactions and consequently higher *T*_B_ values.^24,65^

### 3.5 Hyperthermia Measurements

**Figure 5:**
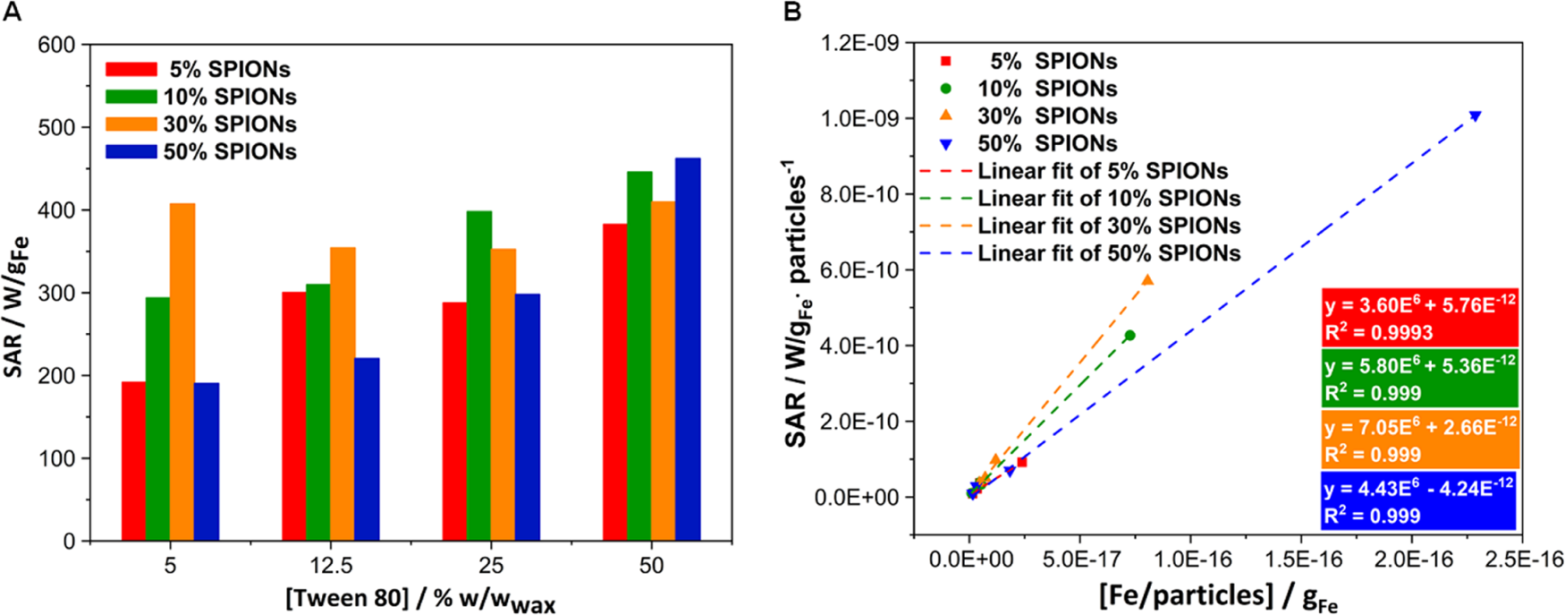
Hyperthermia measurements of 5, 10, 30, and 50% SPION-loaded SLNs at different Tween80 concentrations (5, 12.5, 25, 50% w/w_wax_) using an oscillating magnetic field of 200 Oe and a frequency of 869 kHz: (A) specific absorption rate (SAR, expressed as W·g_Fe_^–1^) values as a function of different surfactant concentrations used during the synthesis and (B) linear response of the SAR per particle as a function of different Fe loadings per particle.

The efficacy of the different mSLN formulations as MH nanoheaters was also evaluated. mSLN formulations were tested at a fixed Fe concentration (0.1 mg_Fe_·mL^–1^), and their capability to increase the temperature of a water solution under an alternating magnetic field (*H* = 200 Oe, *f* = 869 kHz) was measured under adiabatic conditions to minimize heat loss. The formulations prepared at the lowest magnetic loadings (1 and 2% SPIONs) had to be disregarded as the required Fe concentration to generate macroscopic temperature differences could not be attained. It is worth mentioning that all of the measurements were performed under conditions below the limit considered biologically safe (according to the Brezovich criterion).^66^ The heating curves of the mSLN formulations as a function of time are displayed in Figure SI 3. SPION configuration, interparticle spacing, and physical confinement are some of the characteristics that influence the magnetic heating behavior of encapsulated hybrid nanosystems.^16,17,19,31,67,68^ In this confined nanosystem, the obtained SAR values were in the range between 200 and 450 W·g_Fe_^–1^, considered suitable for magnetic hyperthermia applications.^16,69^ The analysis of the SAR obtained for each sample shows a progressive increase of SAR as a function of Tween80 concentration for all SPION contents, except for the 30% series where an initial decrease in SAR is observed up to 25% Tween80. Moreover, as the surfactant percentage increases, the role of magnetic loading becomes less significant. At low Tween80 percentages, particularly at 5%, differences in magnetic loading clearly provide significant differences in performance (expressed in terms of SAR values, Figure 5A). As the percentage of the surfactant increases, these differences become less obvious. In any case, the straightforward idea that higher magnetic loadings lead to better hyperthermia efficiencies is not true in this confined nanosystem. Indeed, samples prepared with the highest magnetic loading (50% SPIONs) do not provide better performances in any of the cases studied. The SAR of the formulations initially increases as the magnetic loading increases to reach a maximum at 30% (10% in the case of 25% Tween80). From there, the efficiency decreases with increasing magnetic loading. A similar behavior has already been reported for other confined magnetic nanosystems and was attributed to a direct effect of confinement through increased dipole–dipole magnetic interactions.^65^ An analysis of the SAR from the point of view of individual mSLNs, accounting for the Fe loading per mSLNs, as proposed elsewhere,^65^ provides a rational explanation showing that the SAR increases linearly with the magnetic loading of individual mSLNs (Figure 5B).^65^ These results are in good agreement with the magnetic properties discussed above and further demonstrate the important role of surfactant concentration in the magnetic performance of the mSLNs, in turn highlighting the amount of Fe per mSLN as a more suitable parameter for understanding the final performance.

### 3.6 Relaxometric Properties and Magnetic Resonance Imaging Studies

Anisotropy and magnetic interactions have been extensively studied to understand the relaxometric behavior of (individual) magnetic nanoparticles. Relaxivity is defined as the concentration (mM) of contrast agent required to modify the relaxation time of water protons in 1 s. It is particularly influenced by the distance between magnetic particles and external water molecules. *T*_2_ and *T*_1_ relaxation times of water protons in the presence of mSLNs were measured at different Fe concentrations (0–0.15 mM), 37 °C, and 1.41 T. As expected, a linear dependence of *T*_2_^–1^ and *T*_1_^–1^ on Fe concentration was found for all mSLN samples, providing an indirect indication of the colloidal stability of the mSLN dispersion, at least during the time frame of the measurements. The corresponding relaxivity values, transversal (*r*_2_) and longitudinal (*r*_1_), were obtained from the linear fitting of *T*_2_^–1^ and *T*_1_^–1^ versus Fe concentration and are outlined in Table SI 3. mSLNs, due to their superparamagnetic character, act predominantly as *T*_2_ contrast agents, and their performance is far superior to commercial *T*_2_ contrast agents such as Resovit (*r*_2_ = 61 mM^–1^·s^–1^) or Feridex (*r*_2_ = 41 mM^–1^·s^–1^).^70^ Regardless of this, longitudinal relaxation (*r*_1_) was also studied in the search for deeper insights into the effect of magnetic loading on confined nanosystems.

**Figure 6:**
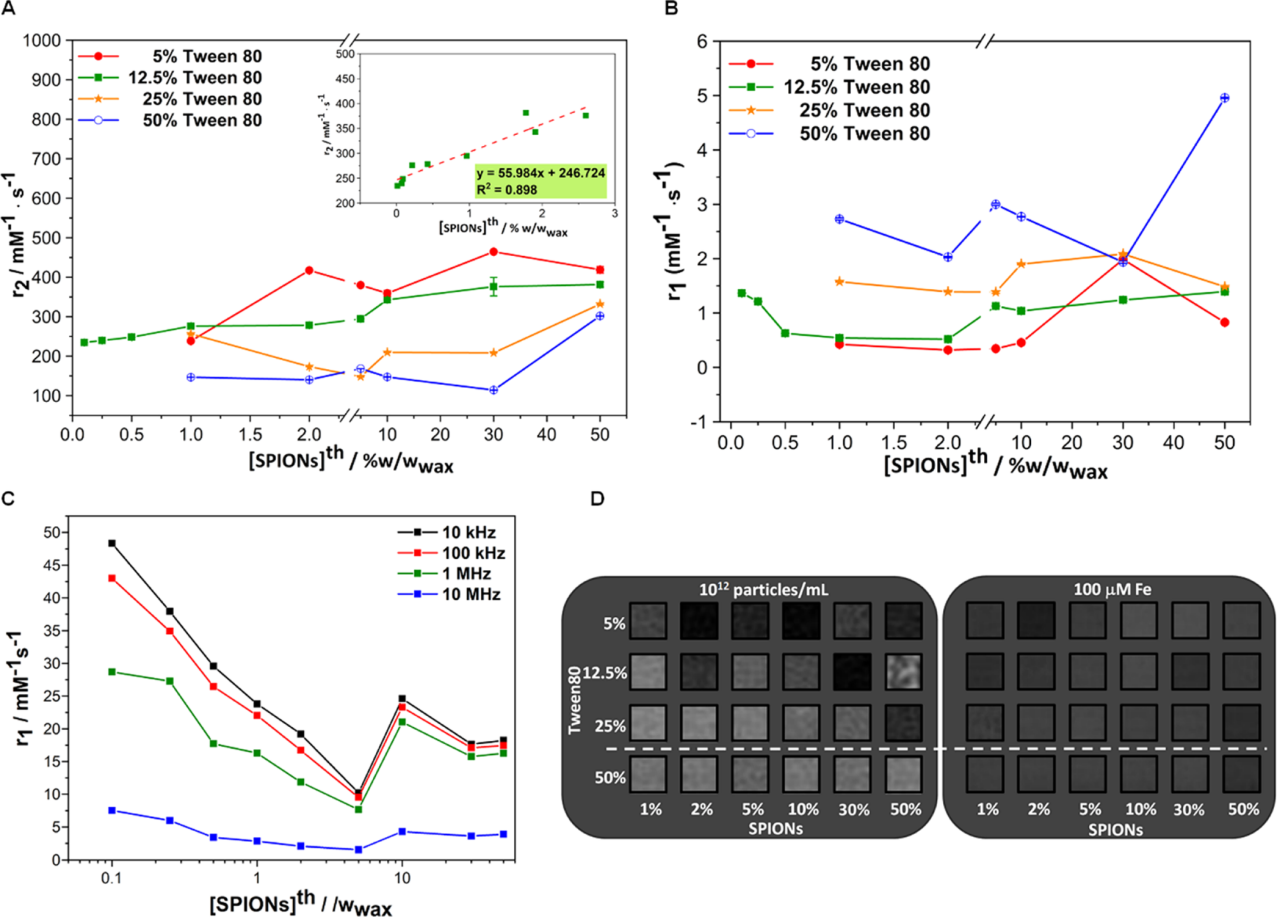
(A) Transversal (*r*_2_) and (B) longitudinal (*r*_1_) relaxivities as a function of the theoretical SPION loading for the mSLNs prepared at different Tween80 concentrations. (C) Longitudinal (*r*_1_) relaxivity as a function of the theoretical SPION loading for the mSLNs prepared at 12.5% w/w_wax_ Tween80 concentration, recorded at different frequencies (from 5 kHz to 10 MHz). *T*_2_-weighted MR phantom images of the different formulations prepared in this work (D) at the same concentration of particles per mL (10^12^ particles·mL^–1^) and (E) at the same iron concentration (100 μM Fe).

Some general conclusions are immediately noticeable when looking at the results (see Figure 6A,B and Table SI 3). First, mSLNs prepared with 50% Tween80 presented *r*_2_ values generally lower than those of the remaining formulations, while their *r*_1_ values were higher. Both observations make sense considering that this series is composed of individual noninteracting magnetic nanoparticles. The *r*_2_ values recorded for these samples are relatively constant and within the range 115–170 mM^–1^·s^–1^, in good agreement with data in the literature for classic nonconfined, noninteracting iron oxide-based contrast agents. *r*_1_ values follow a similar trend, varying within 2–3 mM^–1^·s^–1^ for most of the series, as shown in Figure 6B. Only at the highest magnetic loading, *r*_1_ increases significantly (≈5 mM^–1^·s^–1^), which can be ascribed to the decrease of the lipid shell stabilizing the nanoparticles, leaving the paramagnetic Fe centers more exposed.

Continuing with *r*_2_, the values increase significantly for the rest of series; as the Tween80 concentration decreases, *r*_2_ increases over the whole range of the magnetic loading tested. In these confined nanocomposites, the effect of intra-mSLN magnetic interactions starts to be observed with an increase in *r*_2_ values. As reported by Lee et al., SPIONs embedded in organic matrixes can form clusters, facilitating the *T*_2_ relaxation process displaying, consequently, higher *r*_2_ relaxivity. This can represent a successful strategy to increase magnetic responsiveness while retaining the superparamagnetic characteristics. Each nanoparticle cluster can theoretically be considered a large magnetized sphere with an overall magnetic moment proportional to its size.^71^ Therefore, the larger the size of the aggregates, the larger the *r*_2_ relaxivity. Although this appears to be in general true in this case, a deeper look into the results shows that, for a fixed magnetic loading, the *r*_2_ relaxivity decreases as the Tween80 concentration increases (e.g., 2% SPION, *r*_2_ = 417, 228 and 173 mM^–1^·s^–1^ for 5, 12.5, and 25% Tween80, respectively). On the one hand, this is an unexpected observation as the surfactant concentration directly correlates to Fe concentration (Figure 3) and thus the opposite trend (an increase of *r*_2_ with Tween/Fe) would be likely as larger intra-SLN interactions would be in place as the magnetic cargo increases. This trend is actually observed in the case of the *r*_1_ relaxivity, where at a fixed SPION loading, an increase in Tween80 is associated with an increase in *r*_1_. On the other hand, by increasing the surfactant concentration, the size of the mSLNs decreases and the number of mSLNs increases. Even though the concentration of SPIONs also increases, the number of SPION per mSLN will decrease (the increase in the number of mSLNs is more pronounced than the increase in SPION concentration), with the final outcome being that the whole system becomes less interacting (Scheme S1 and Table SI 4). This second scenario explains well the behavior observed in both magnetic hyperthermia and *r*_2_ relaxivity and highlights the importance of a parameter that is rarely measured in nanoformulations: the number of particles per mL. In the case of the series at 5, 12.5, and 25% Tween80, *r*_2_ values are not constant, but an upward trend is observed within the series, pointing to stronger magnetic interactions. In *r*_1_, this effect is less obvious like the magnetic interaction effect. To further explore these effects in a wider magnetic loading range, the series prepared at 12.5% Tween80 was extended to lower SPION loadings (0.1–0.25 and 0.5% w/w_wax_). Even in these low magnetic content samples, the relaxivities (both *r*_1_ and *r*_2_) follow the same observed trend. In fact, a linear relationship was observed along the series between SPION loadings and *r*_2_ values (Figure 6A, inset). The *r*_1_, however, shows a strong decrease at very low magnetic loadings (0.1–0.5% w/w_wax_). Overall, the surfactant seems to play an unexpected key role in the relaxometric properties (both longitudinal and transversal) of these confined systems. Higher levels of Tween80 present a deleterious effect on *r*_2_ properties. As the proportion of Tween80 decreases, the *r*_2_ increases. There is a limit, however, not directly related to the relaxometric performance but to the polydispersity and colloidal stability of the samples. As shown above, mSLNs prepared without the surfactant do not reach acceptable PDI levels for biomedical applications.

An analysis of the *r*_2_ relaxivity similar to the one done for the SAR, relating the performance per particle to the Fe content per particle, also shows interesting results (Figure SI 4). The different series prepared fit reasonably well to linear regression. The series at 5 and 12.5% Tween80 are the ones in which an increase in magnetic loading brings a stronger change in the relaxivity per particle (Figure SI 4A,B, respectively). The effect is weaker in the 25% Tween80 series (Figure SI 4C) and weakest in the 50% Tween80 (Figure SI 4D), which correlates with the fact that the Fe per particle of the 50% series does not change with the initial SPION loading (individual noninteracting core@shell nanoparticles).

To dig a bit deeper into the relaxometric properties of these confined magnetic systems, the ^1^H spin–lattice relaxation rates of the extended series at 12.5% Tween80 were measured in the frequency range from about 5 kHz to 10 MHz. The relaxation process turned out to fit a single exponential function for all samples at all frequencies (see Figure SI 5). The *r*_1_ relaxation rate of these samples at the low frequency range tested followed the expected qualitative trend for the lower magnetic loadings (0–10%), with an observed increase in *r*_1_. For the highest loadings (30 and 50%), this trend is lost and *r*_1_ decreases (Figure SI 6A, orange and light green squares, respectively). A look at the calculated *r*_1_ relaxivity values brings more unexpected results. The relaxivity monotonically decreases with increasing SPION concentration from 0.1 to 5% (Figure 6C). This effect might be considered surprising, as one expects the relaxivity of the mSLNs to increase with increasing SPION loading. Then, for the 10% concentration, the relaxivity somewhat increases, to decrease again for higher concentrations. This effect results from a competition between the increasing concentration of the paramagnetic species that leads (by itself) to a higher relaxivity and faster electronic relaxation (caused by stronger interactions between the paramagnetic centers for higher concentrations) that leads to lower relaxivity as the electronic relaxation acts as a source of modulation of the proton–electron dipole–dipole interactions.

*T*_2_-weighted MR images of the mSLN formulations were then acquired at 3.0 T to corroborate the obtained relaxometric data. Taking into account that each sample is different in terms of magnetic loading and concentration, two different approaches were followed to image the formulations. In the first one, phantoms of all samples were prepared in water at the same particle concentration (10^12^ particles·mL^–1^, Figure 6D). In the second one, phantoms were prepared at the same Fe concentration (100 μM, Figure 6E). In the first case, having the same number of particles per sample, the concentration of iron increases (thus the contrast should increase) from sample to sample only because the magnetic loading per particle increases. In the second case, with the concentration of Fe being constant, the contrast generated should be constant if potential magnetic interaction effects are not in place.

Looking first at the images acquired at the same number of particles per sample, as before, results show clearly that samples prepared at the 50% Tween80 content are different from the rest as, under the imaging conditions used, none of these samples were capable of generating significant signals (in accordance with the lower *r*_2_ values recorded before). Then, also in accordance with relaxivity data, a signal enhancement is observed as the content of Tween80 decreases (moving up the rows). As in the case of transversal relaxivity, this is opposite of the expected effect as the content of Tween80 is directly related to the Fe content and thus, in theory, to increased magnetic character and performance. However, as discussed above, this behavior probably has to do with the effect of a higher number of particles per mL overpowering the effect of increased SPION content. Focussing on the same series, contrast generation does not seem to be related to magnetic loading either. Only at high Tween80 concentrations (25%) is a linear correlation between loading and contrast generation (see Figure SI 7). At 5 and 12.5% Tween80, the contrast changes from sample to sample without trend. Regarding the images acquired at the same Fe concentration, results do not show the same contrast from all the samples, as expected in the absence of confinement effects. In the series prepared at a 25% Tween80 content, the contrast increases at high magnetic loadings, consistent with higher dipolar effects as the inter-SPION distance within the same particle decreases. In the other two samples, 5 and 12.5% Tween80, a higher magnetic loading, and thus, a closer distance between SPIONs do not translate into higher contrast. At 5% Tween80, the contrast increases initially from 1 to 2% SPIONs but then decreases significantly. At 12.5% Tween80, the contrast decreases from 1 to 10% and then recovers and platoons from 20%. These results are a clear example of the complexity of these systems and demonstrate that predicting or even explaining the behavior of these multifunctional systems is not easy.

## 4 Conclusions

The general requirements for the successful behavior of magnetic nanomaterials in vivo were established many years ago, and they are well understood. The same is more or less true for the influence that these magnetic properties have on the performance of magnetic nanomaterials in magnetism-based biomedical applications such as MRI and MH, as long as we deal with individual noninteracting systems. However, the situation with interacting systems, whether they are confined systems such as the one subject of this work, or materials where species of different magnetic nature are in close proximity, still represents a challenge as the properties of these systems are difficult to predict and even explain. In this work, we have systematically studied the behavior of a confined magnetic nanocomposite in MRI and MH, trying to relate observed performances to magnetic phenomena occurring in the particles. The results demonstrate how complex these systems are and how difficult it is to find a relationship between physicochemical and magnetic properties and performance. Our findings highlight the crucial role of surfactant concentration in the final performance of the magnetic nanocomposites, which resulted to be even more relevant that the magnetic loading itself. However, only when considering the systems from a single nanocomposite point of view can some light be partially shed on the obtained results, at least for the MH data. For MRI data, some clear correlations were found using this approach, but they are not yet fully understood. The multiplex correlation between different key factors, namely, Fe content, surfactant concentration, and mSLN concentration and number, determine the potential intra- and interparticle dipole–dipole magnetic interactions, which might be playing a major role in the final mSLN performance. Initially, surprising results like the big influence of surfactant concentration on the final functional properties can be understood from an individual nanocomposite view, from the influence of the surfactant on the final size, and thus number, of nanocomposites. Thus, these preliminary results open the door to further fundamental studies where a combination of theoretical simulations and experimental results can offer a full description of the physical phenomena that govern the theranostic performance of these systems in the biomedical field.

## Supporting Information Available

Physicochemical characterization of hydrophobic SPIONs (Figure SI 1 and Table SI 1); SPIONs’ encapsulation efficiency within mSLN series (Table SI 2), blocking temperature of mSLN series (Figure SI 2), heating kinetics curves of mSLN series (Figure SI 3), scheme of inter-SPION distances differences between the mSLN series (Scheme SI 1), longitudinal and transversal relaxivity values of the mSLN series (Table SI 3), correlation between surfactant concentration and mSLN series physicochemical parameters (Table SI 4), linear fitting of transversal relaxivity within mSLN series (Figure SI 4), ^1^H magnetization curves of mSLN series (Figure SI 5), ^1^H spin–lattice relaxation rates and longitudinal relaxivity of mSLN series (Figure SI 6), and linear fitting of contrast intensity signals within mSLN series (Figure SI 7) (PDF)

## Abbreviations

AMF: alternating magnetic field
AP: aqueous phase
COD: Crystallography Open Database
*D*_h_: hydrodynamic diameter
DLS: dynamic light scattering
FDA: Food and Drug Administration
*H*_c_: coercivity
MH: magnetic hyperthermia
MRI: magnetic resonance imaging
*M*_s_: magnetic saturation
mSLNs: magnetic solid lipid nanoparticles
NTA: nanoparticle tracking analysis
OA: oleic acid
OP: organic phase
PDI: polydispersion index
PP: polypropylene
*r*_2_: transverse relaxivity
*r*_1_: longitudinal relaxivity
SAR: specific absorption rate
SLNs: solid lipid nanoparticles
SQUID-VSM: superconducting quantum interference-vibrating sample magnetometer
SPIONs: superparamegnetic iron oxide nanoparticles
*T*_B_: blocking temperature
*T*_Bmin_: blocking temperature minimum
TEM: transmission electron microscopy
*T*_2_: transverse relaxation time
*T*_1_: longitudinal relaxation time
TGA: thermogravimetric analysis
ZFC–FC: zero-field-cooled–field-cooled
