# Supplementary material for: Insights into the Effect of Magnetic Confinement on the Performance of Magnetic Nanocomposites in Magnetic Hyperthermia and Magnetic Resonance Imaging
Source: ACS Appl Nano Mater. 2022 Nov 7;5(11):16462–74. doi: 10.1021/acsanm.2c03537 (PMC9778729; doi:10.1021/acsanm.2c03537)
Supplement: Supplementary file 1 — an2c03537_si_001.pdf [file an2c03537_si_001.pdf]

Insights into the effect of magnetic confinement on the performance of magnetic nanocomposites in magnetic hyperthermia and magnetic resonance imaging

Stefania Scialla[1, ϕ], Nuria Genicio[1], Beatriz Brito[1,2,3], Malgorzata Florek-Wojciechowska[4], Graeme J. Stasiuk[2], Danuta Kruk[4], Manuel Bañobre-López[1], Juan Gallo[1*]*

[1]Advanced (magnetic) Theranostic nanostructures Lab, International Iberian Nanotechnology Laboratory, Av. Mestre José Veiga s/n 4715-330 Braga, Portugal

[2]Department of Imaging Chemistry and Biology, School of Biomedical Engineering and Imaging Sciences, King's College London, Strand, London, UK, SE1 7EH.

[3]School of Life Sciences, Faculty of Health Sciences, University of Hull, Cottingham Road, Hull, UK, HU6 7RX.

[4]Department of Physics and Biophysics, Faculty of Food Science, University of Warmia & Mazury in Olsztyn, Oczapowskiego 4, 10-719 Olsztyn

Corresponding author's email: Juan.gallo@inl.int; Manuel.banobre@inl.int

7 Supporting Information Figures

1 Supporting Information Scheme

4 Supporting Information Tables

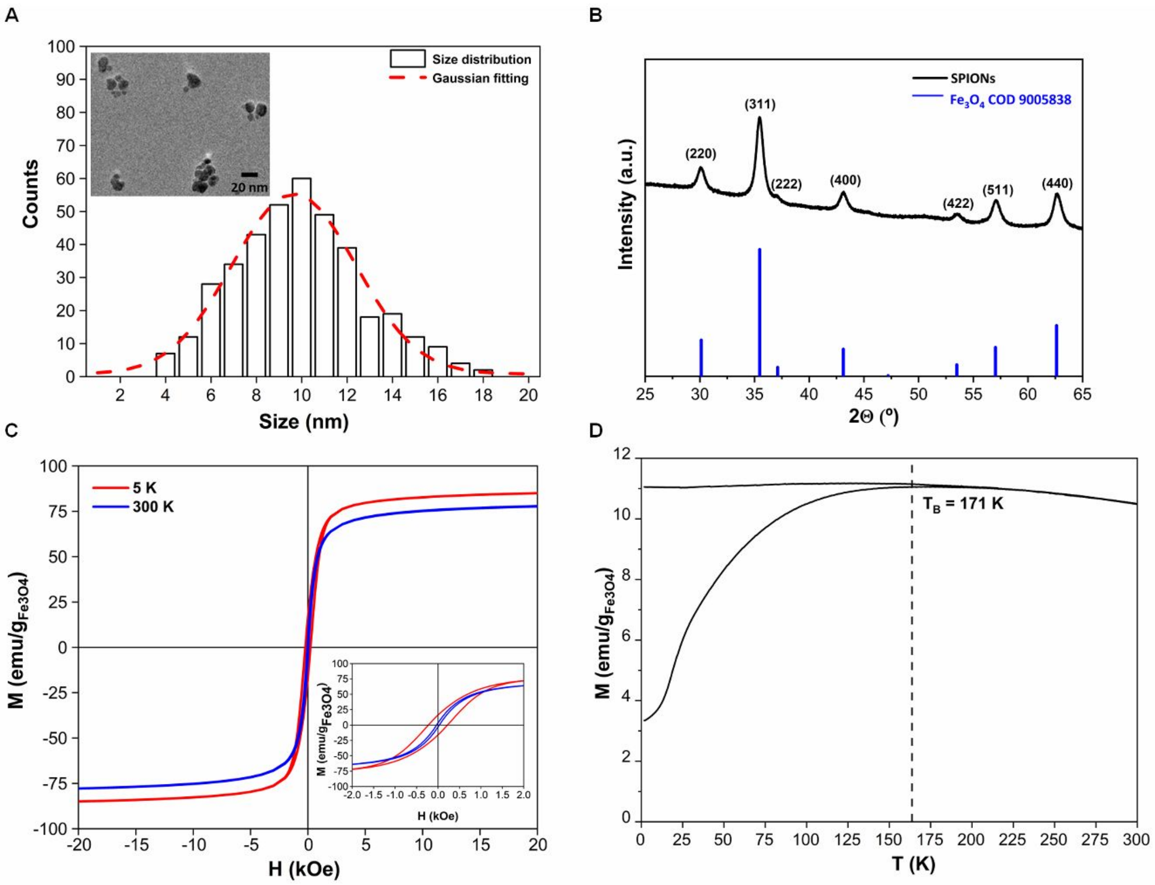

Figure SI 1 Physico-chemical characterization of hydrophobic SPIONs: **(A)** size distribution from TEM micrographs analysis (total count > 300) fitted to a Gaussian distribution [SPIONs TEM micrograph insert, Mag 500 kX - scale bar represents 20 nm]; **(B)** indexed powder X-ray diffraction pattern compared to Fe_3O_4 (COD 96-900-5838, blue peaks); **(C)** hysteresis loop in the applied magnetic field range from -20 to +20 kOe at 300 K (blue line) and 5K (red line); **(D)** ZFC-FC magnetization curves recorded over the temperature range 2 – 300 K and under an applied magnetic field of 100 Oe

***Table SI 1** Summary of the main physico-chemical parameters of the hydrophobic SPIONs*

	**Size$_{TEM}$** (nm)	**[Fe_3O_4]$_{TGA}$** (%)	**[OA]$_{TGA}$** (%)	**M_s** (emu/g)[a]	**T_B** (K)[b]
SPIONs	9.7±0.1	85	15	67	170

[a]Saturation Magnetization (M_s) measured at 300 K expressed as emu per g of vacuum-dried *SPIONs*
[b]Blocking temperature (T_B) extrapolated from the ZFC-FC curve ranging from 2 and 300 K, with B= 100 Oe

Table SI 2 *Experimental SPIONs (SPIONs^{ex}) concentration and encapsulation efficiency (EE_{SPIONs}) in the different mSLN formulations.*

**mSLNs**	**Tween 0** (% w/w_{wax})		**Tween 5** (% w/w_{wax})		**Tween 12.5** (% w/w_{wax})		**Tween 25** (% w/w_{wax})		**Tween 50** (% w/w_{wax})	
**SPIONsth** (% w/w_{wax})	**SPIONs^{ex}** (% w/w_{wax})	**EE_{SPIONs}** (%)	**SPIONs^{ex}** (% w/w_{wax})	**EE_{SPIONs}** (%)	**SPIONs^{ex}** (% w/w_{wax})	**EE_{SPIONs}** (%)	**SPIONs^{ex}** (% w/w_{wax})	**EE_{SPIONs}** (%)	**SPIONs^{ex}** (% w/w_{wax})	**EE_{SPIONs}** (%)
**0**	-	-	-	-	-	-	-	-	-	
**1**	-0.01±(-1.20)	100	0.09±0.40	88	0.22±0.10	70	0.14±0.31	81	0.20±0.25	72
**2**	0.02±(-0.25)	100	0.15±0.15	89	0.43±0.28	71	0.30±0.16	79	0.41±0.12	72
**5**	0.01±1.19	99	0.47±0.07	87	0.96±0.16	73	0.90±0.10	75	1.13±0.07	69
**10**	0.06±2.69	99	1.11±0.26	85	1.90±0.19	74	2.06±0.07	72	2.22±0.12	69
**30**	0.33±0.20	99	0.29±0.49	99	2.60±0.18	88	6.68±0.18	69	6.58±0.18	70
**50**	0.47±0.53	99	0.50±0.93	99	1.78±0.34	95	5.87±0.18	84	10.8±0.3	70

SPIONsth: theorical amount of magnetite expressed as % w/w_{wax}
SPIONs^{ex}: experimental amount of magnetite calculated from the corresponding Fe content quantified by ICP-OES and expressed as % w/w_{wax}
EE_{SPIONs}: encapsulation efficiency of magnetite

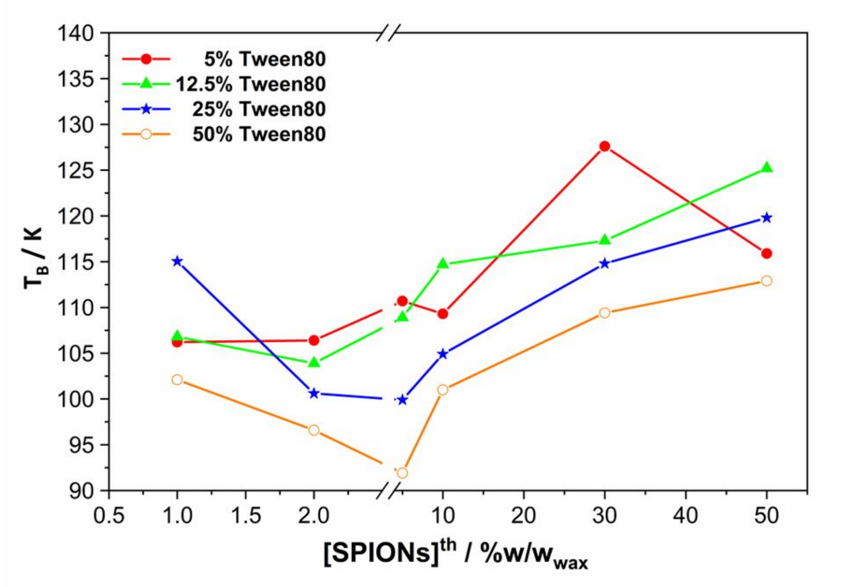

Figure SI 2 *Blocking temperature (T_B) as function of the theorical Fe_3O_4 loading for the mSLN series prepared at the different Tween80, measured in the* range of 2 – 300 K and under an applied magnetic field of 100 Oe.

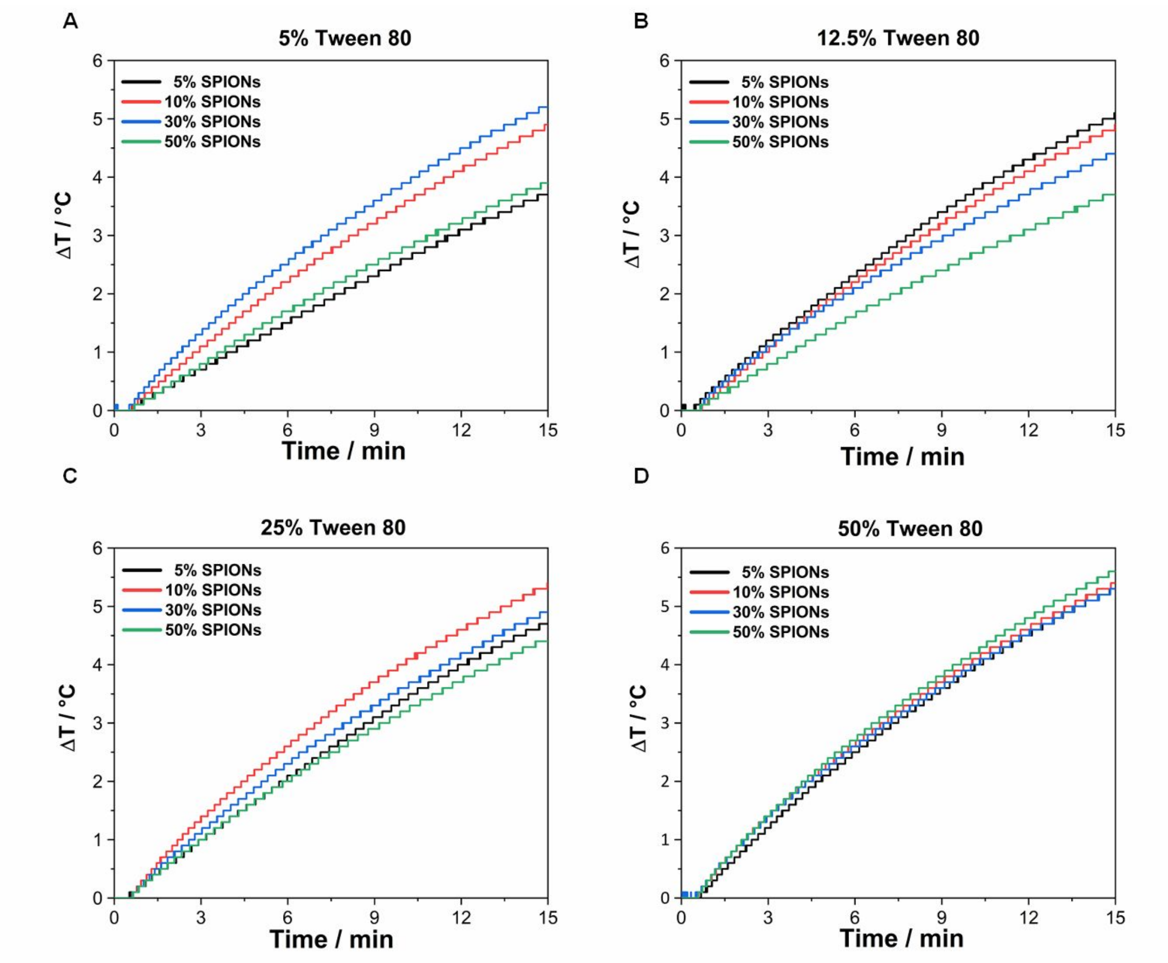

Figure SI 3 *Heating kinetics curves of 5, 10, 30 and 50% SPIONs-loaded SLNs prepared at 5%* ***(A)****, 12.5%* ***(B)****, 25%* ***(C)*** *and 50%* ***(D)*** *Tween80, recorded under an oscillating magnetic field at 200 Oe and a frequency of 869 kHz.*

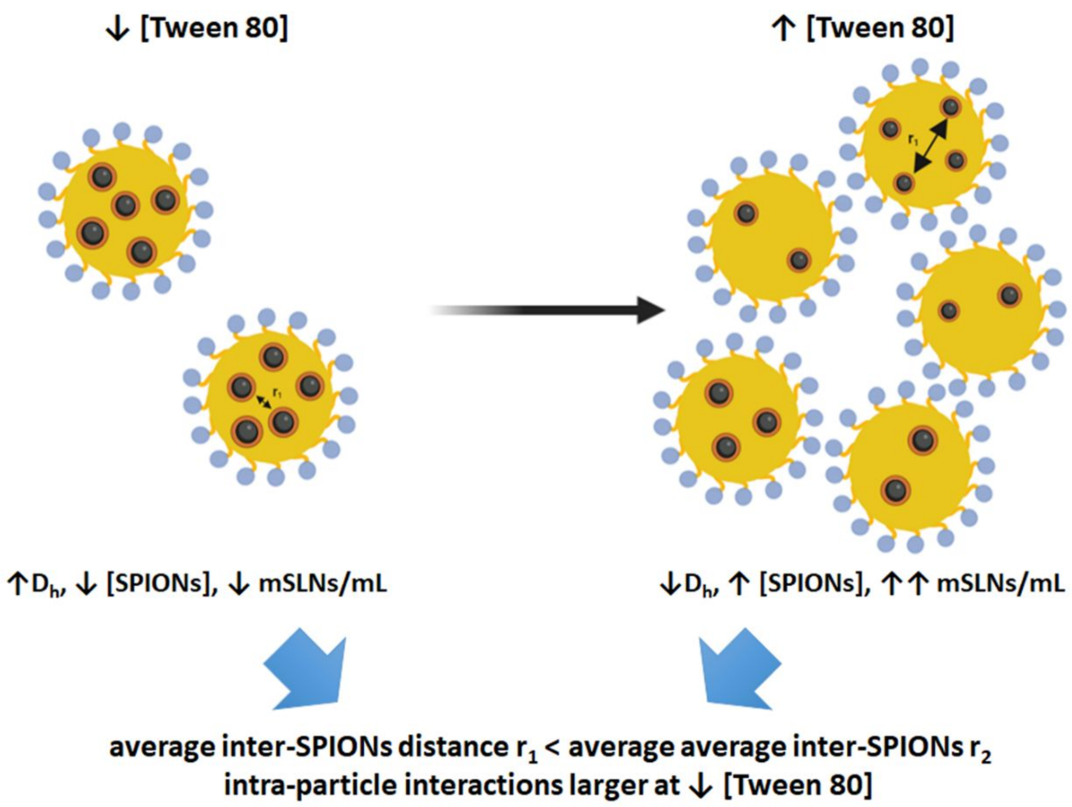

Scheme SI 1. *Differences in inter-SPION distances between the mSLNs prepared under different Tween80 concentrations.*

Table SI 3 *Longitudinal (r_1) and transversal (r_2) relaxivity values of the mSLN formulations measured at different Fe concentrations (0 – 0.15 mM) by using a relaxometer operating at 1.41 T, 60 MHz and 37 °C.*

**mSLNs**	**Tween 5** (% w/w_{wax})		**Tween 12.5** (% w/w_{wax})		**Tween 25** (% w/w_{wax})		**Tween 50** (% w/w_{wax})	
**SPIONsth** (% w/w_{wax})	r_1 ($mM^{-1}\cdot s^{-1}$)	r_2 ($mM^{-1}\cdot s^{-1}$)	r_1 ($mM^{-1}\cdot s^{-1}$)	r_2 ($mM^{-1}\cdot s^{-1}$)	r_1 ($mM^{-1}\cdot s^{-1}$)	r_2 ($mM^{-1}\cdot s^{-1}$)	r_1 ($mM^{-1}\cdot s^{-1}$)	r_2 ($mM^{-1}\cdot s^{-1}$)
**0.1**			1.4 ± 0.01 (R^2 = 0.9980)	234.7 ± 0.2 (R^2 = 0.9972)				
**0.25**			1.2 ± 0.01 (R^2 = 0.9984)	239.5 ± 0.4 (R^2 = 0.9981)				
**0.5**			0.62 ± 0.002 (R^2 = 0.9965)	248.3 ± 0.2 (R^2 = 0.9992)				
**1**	0.42 ± 0.014 (R^2 = 0.9892)	238.5 ± 0.6 (R^2 = 0.9851)	0.54 ± 0.01 (R^2 = 0.9881)	276.0 ± 1.6 (R^2 = 0.9992)	1.57 ± 0.01 (R^2 = 0.9906)	256.3 ± 1.8 (R^2 = 0.9895)	2.73 ± 0.02 (R^2 = 0.9746)	146.7 ± 0.1 (R^2 = 0.9806)
**2**	0.32 ± 0.001 (R^2 = 0.9908)	417.2 ± 0.8 (R^2 = 0.9902)	0.52 ± 0.01 (R^2 = 0.9978)	278.4 ± 0.2 (R^2 = 0.9982)	1.39 ± 0.004 (R^2 = 0.9964)	173.5 ± 0.05 (R^2 = 0.9986)	2.03 ± 0.01 (R^2 = 0.9927)	140.3 ± 0.4 (R^2 = 0.9930)
**5**	0.35 ± 0.02 (R^2 = 0.9701)	379.9 ± 0.7 (R^2 = 0.9986)	1.13 ± 0.01 (R^2 = 0.9824)	294.6 ± 0.6 (R^2 = 0.9999)	1.38 ± 0.022 (R^2 = 0.9933)	147.9 ± 0.6 (R^2 = 0.9991)	3.00 ± 0.02 (R^2 = 0.9974)	168.4 ± 0.2 (R^2 = 0.9980)
**10**	0.46 ± 0.02 (R^2 = 0.9920)	359.2 ± 7.2 (R^2 = 0.9933)	1.04 ± 0.002 (R^2 = 0.9919)	342.8 ± 0.3 (R^2 = 0.9979)	1.9 ± 0.01 (R^2 = 0.9985)	209.5 ± 0.3 (R^2 = 0.9996)	2.77 ± 0.004 (R^2 = 0.9990)	147.5 ± 0.6 (R^2 = 0.9996)
**30**	1.99 ± 0.01 (R^2 = 0.9940)	464.6 ± 2.8 (R^2 = 0.9937)	1.24 ± 0.02 (R^2 = 0.9965)	375.9 ± 23.3 (R^2 = 0.9971)	2.09 ± 0.01 (R^2 = 0.9979)	208.4 ± 0.8 (R^2 = 0.9990)	1.94 ± 0.02 (R^2 = 0.9951)	114.2 ± 0.5 (R^2 = 0.9989)
**50**	0.83 ± 0.01 (R^2 = 0.9986)	418.8 ± 8.4 (R^2 = 0.9967)	1.39 ± 0.01 (R^2 = 0.9976)	381.2 ± 6.7 (R^2 = 0.9978)	1.48 ± 0.01 (R^2 = 0.9983)	331.9 ± 2.1 (R^2 = 0.9935)	4.96 ± 0.01 (R^2 = 0.9984)	301.9 ± 0.2 (R^2 = 0.9988)

Table SI 4 *Correlation between Tween80 concentration and physico-chemical parameters (experimental SPIONs loading, hydrodynamic diameter and nanoparticles concentration) for all mSLN formulations.*

	**mSLNs**														
	**Tween 0** (% w/w$_{wax}$)			**Tween 5** (% w/w$_{wax}$)			**Tween 12.5** (% w/w$_{wax}$)			**Tween 25** (% w/w$_{wax}$)			**Tween 50** (% w/w$_{wax}$)		
**SPIONs$_{th}$** [a]	**SPIONs$_{ex}$** [b]	**D$_h$**[c]	**[mSLN]**[d]	**SPIONs$_{ex}$** [b]	**D$_h$**[c]	**[mSLN]**[d]	**SPIONs$_{ex}$** [b]	**D$_h$**[c]	**[mSLN]**[d]	**SPIONs$_{ex}$** [b]	**D$_h$**[c]	**[mSLN]**[d]	**SPIONs$_{ex}$** [b]	**D$_h$**[c]	**[mSLN]**[d]
**0**	-	210±16	$4.1E^{12}\pm5.5E^{11}$	-	185±12	$5.4E^{12}\pm4.2E^{11}$	-	162±16	$1.8E^{14}\pm1.1E^{13}$	-	138±14	$3.9E^{8}\pm2.5E^{7}$	-	65±1	$4.2E^{13}\pm3.9E^{12}$
**1**	0.09±0.40	240±24	$8.6E^{12}\pm1.3E^{12}$	0.09±0.40	181±18	$1.0E^{13}\pm1.8E^{11}$	0.22±0.10	181±25	$2.4E^{14}\pm1.9E^{13}$	0.14±0.31	94±6	$7.6E^{14}\pm3.6E^{13}$	0.20±0.25	39±1	$1.2E^{15}\pm9.7E^{13}$
**2**	0.15±0.15	110±17	$4.9E^{12}\pm2.3E^{11}$	0.15±0.15	282±41	$4.8E^{12}\pm3.5E^{11}$	0.43±0.28	163±55	$1.1E^{13}\pm6.2E^{11}$	0.30±0.16	128±5	$2.3E^{14}\pm1.4E^{13}$	0.41±0.12	61±5	$3.3E^{14}\pm3.2E^{13}$
**5**	0.47±0.07	97±10	$4.9E^{12}\pm2.6E^{11}$	0.47±0.07	203±56	$1.1E^{13}\pm8.8E^{11}$	0.96±0.16	141±41	$1.3E^{14}\pm1.7E^{13}$	0.90±0.10	92±10	$3.3E^{14}\pm2.9E^{12}$	1.13±0.07	42±1	$1.3E^{14}\pm2.7E^{12}$
**10**	1.11±0.26	168±1	$4.3E^{12}\pm5.7E^{11}$	1.11±0.26	144±6	$8.9E^{12}\pm2.2E^{11}$	1.90±0.19	158±36	$1.7E^{14}\pm6.9E^{12}$	2.06±0.07	110±1	$2.3E^{14}\pm1.2E^{13}$	2.22±0.12	41±1	$1.1E^{15}\pm8.6E^{13}$
**30**	0.29±0.49	175±21	$1.1E^{13}\pm4.0E^{11}$	0.29±0.49	137±2	$1.6E^{13}\pm1.3E^{12}$	2.60±0.18	164±54	$2.1E^{13}\pm1.2E^{12}$	6.68±0.18	102±11	$5.4E^{14}\pm3.6E^{13}$	6.58±0.18	61±2	$7.9E^{14}\pm5.6E^{13}$
**50**	0.50±0.93	176±3	$1.2E^{13}\pm1.3E^{12}$	0.50±0.93	122±2	$1.7E^{13}\pm3.8E^{11}$	1.78±0.34	100±30	$7.5E^{12}\pm7.5E^{11}$	5.87±0.18	87±15	$2.3E^{14}\pm8E^{13}$	10.8±0.3	50±2	$2.0E^{15}\pm1.0E^{14}$

[a] theorical amount of magnetite expressed as % w/w$_{wax}$
[b] experimental amount of magnetite calculated from the corresponding Fe content quantified by ICP-OES and expressed as % w/w$_{wax}$
[c] hydrodynamic diameter expressed in nm
[d] mSLN concentration expressed as particles·mL^{-1}

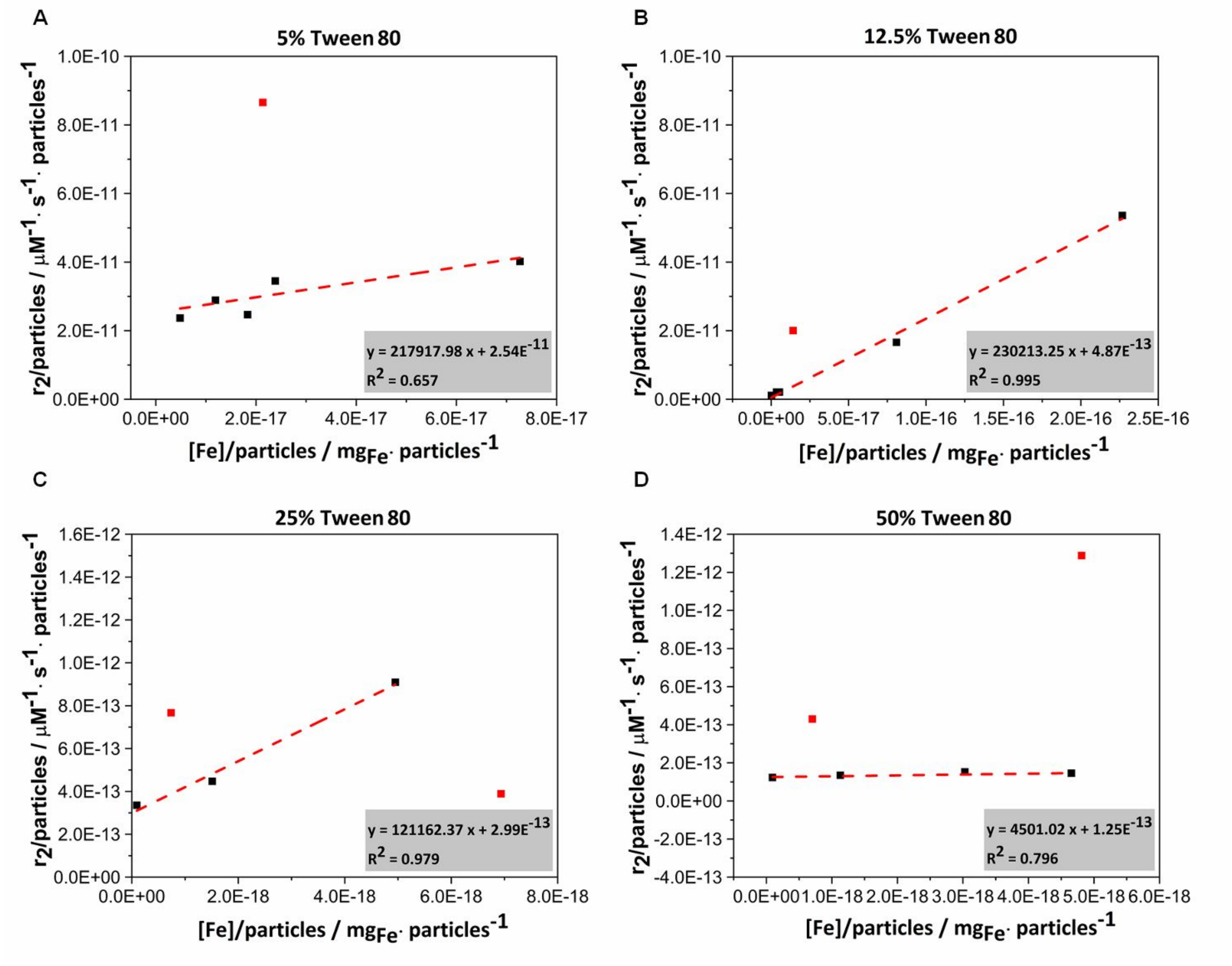

Figure SI 4. *Linear fitting of transversal (r_2) relaxivity per number of particles versus mass of Fe content per number particle for mSLN formulations prepared at 5%* ***(A)****, 12.5%* ***(B)****, 25%* ***(C)*** *and 50%* ***(D)*** *Tween80 concentration.*

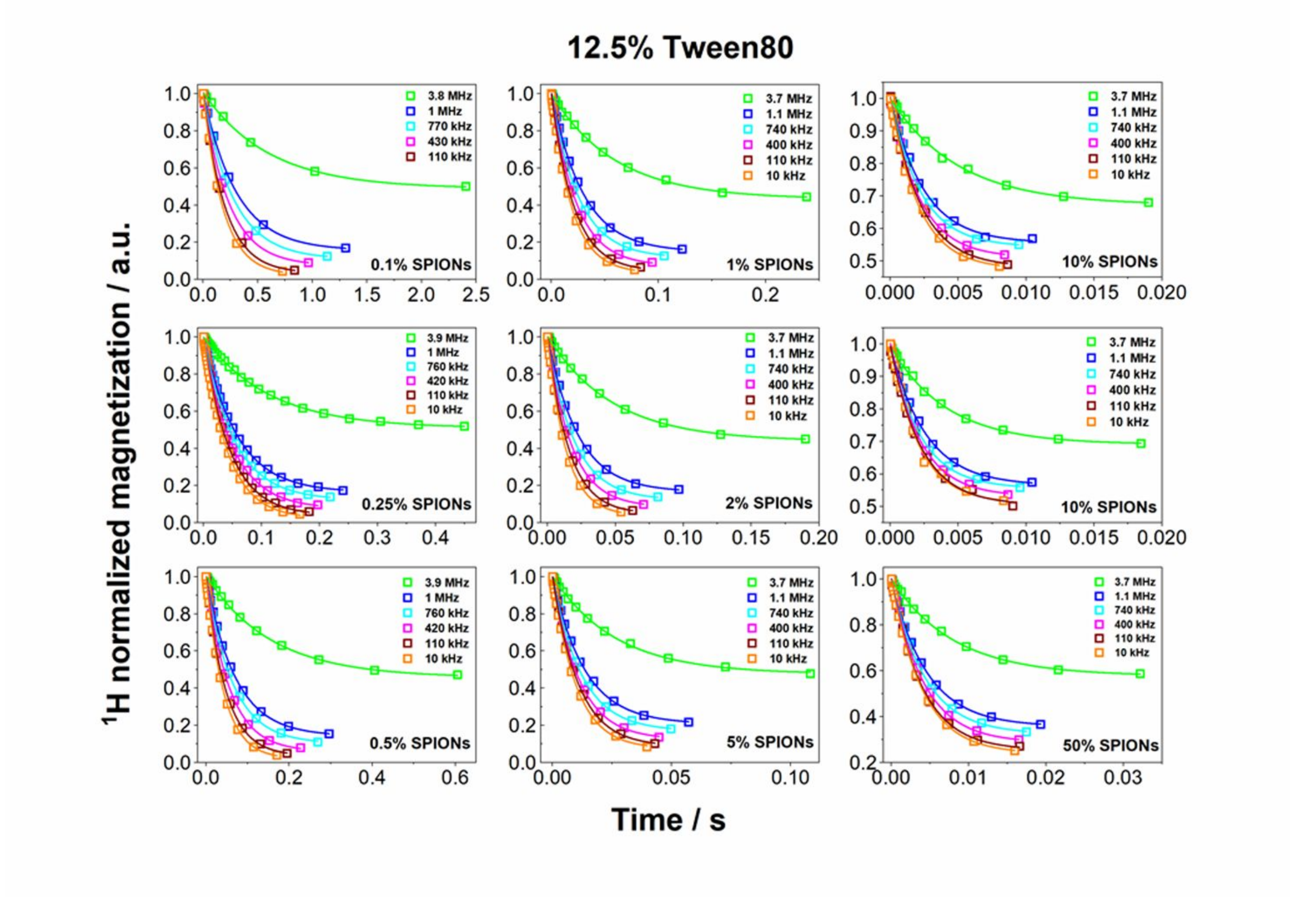

Figure SI 5. ^{1}H magnetization curves of mSLN series (from 0.1 to 50% SPIONs) prepared at 12.5% Tween80 at different resonance frequencies (from 10 kHz to 4 MHz). Solid lines – single exponential fits.

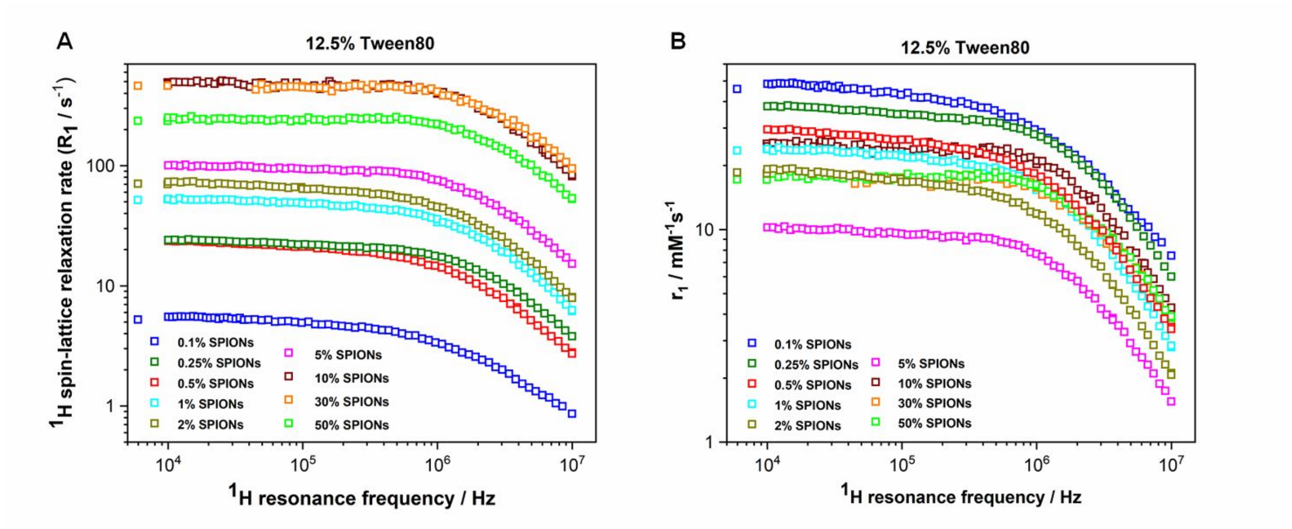

Figure SI 6 (A) ^{1}H spin-lattice relaxation rates (R_1, s^{-1}) and **(B)** longitudinal relaxivity values (r_1, mM^{-1}·s^{-1}) for mSLN formulations (from 0.1 to 50% SPIONs) prepared at 12.5% Tween80.

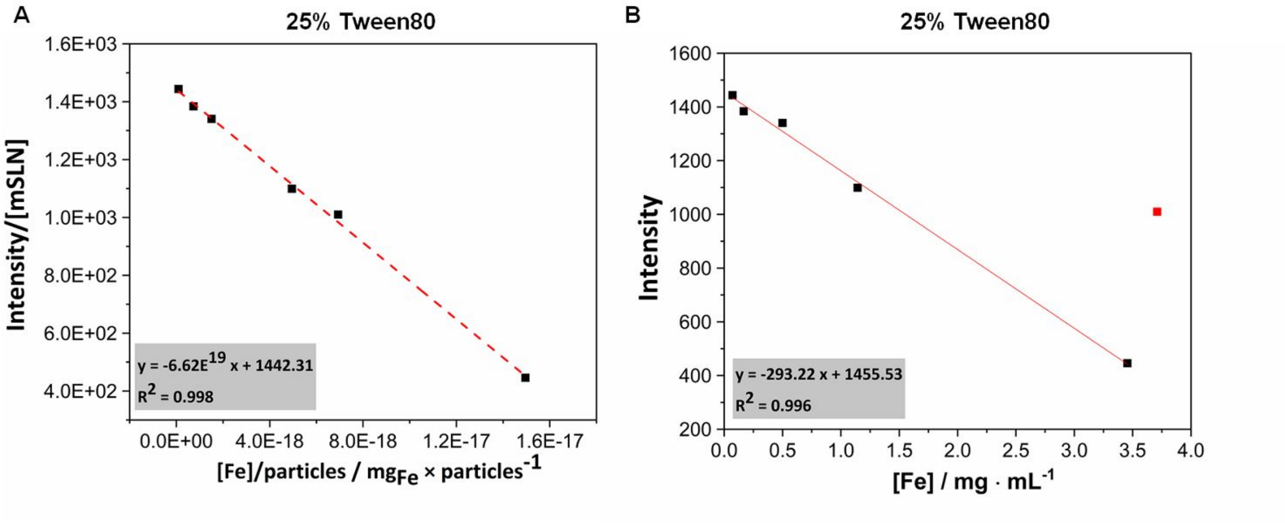

Figure SI 7. Linear fitting of contrast intensity signal generated by 25% Tween80 mSLN formulations versus Fe content per particle extrapolated from T_2-weighted MR images acquired at 3.0 T.
